# How large B-factors can be in protein crystal structures

**DOI:** 10.1186/s12859-018-2083-8

**Published:** 2018-02-23

**Authors:** Oliviero Carugo

**Affiliations:** 1 Department of Structural and Computational Biology, University of Vienna, Campus Vienna Biocenter 5, A-1030 Vienna, Austria; 2 Department of Chemistry, University of Pavia, viale Taramelli 12, I-27100 Pavia, Italy

**Keywords:** Atomic displacement parameter, B-factor, Crystal structure, Matthews coefficient, Protein structure validation

## Abstract

**Background:**

Protein crystal structures are potentially over-interpreted since they are routinely refined without any restraint on the upper limit of atomic B-factors. Consequently, some of their atoms, undetected in the electron density maps, are allowed to reach extremely large B-factors, even above 100 square Angstroms, and their final positions are purely speculative and not based on any experimental evidence.

**Results:**

A strategy to define B-factors upper limits is described here, based on the analysis of protein crystal structures deposited in the Protein Data Bank prior 2008, when the tendency to allow B-factor to arbitrary inflate was limited. This B-factor upper limit (B_max) is determined by extrapolating the relationship between crystal structure average B-factor and percentage of crystal volume occupied by solvent (pcVol) to pcVol =100%, when, ab absurdo, the crystal contains only liquid solvent, the structure of which is, by definition, undetectable in electron density maps.

**Conclusions:**

It is thus possible to highlight structures with average B-factors larger than B_max, which should be considered with caution by the users of the information deposited in the Protein Data Bank, in order to avoid scientifically deleterious over-interpretations.

**Electronic supplementary material:**

The online version of this article (10.1186/s12859-018-2083-8) contains supplementary material, which is available to authorized users.

## Background

Although drastically reduced, atomic movements are possible in crystals, even at low temperature. In crystal structures, oscillation amplitudes of the atoms around their equilibrium positions are monitored by B-factors, which are related to the root-mean-square amplitude of oscillation (*u*) of the atoms around their equilibrium position by the following equation:

1 $$ B=8{\pi}^2{u}^2 $$

The experimental fingerprint of the B-factors is due to their influence on the atomic form factor according to

2 $$ f={f}_o\bullet \mathit{\exp}\left(-\frac{B\bullet {\mathit{\sin}}^2\theta }{\lambda^2}\right) $$

where *f*_*o*_ is the atomic form factor at *B* = 0 Å^2^, *θ* is the diffraction angle, and *λ* is the X-ray wavelength. It is apparent that the scattering power of an atom diminishes as fast as the B-factor increases.

In reality, B-factors do not depend only on the amplitudes of the atomic oscillations around the equilibrium positions but also on other factors. Recently, Kuzmanic and co-workers observed, based on molecular dynamics simulations, that conformational averaging and inadequate treatment of correlated motions considerably influence the estimations of microscopic heterogeneity via B-factors [1]. In a typical crystal structure, B-factors may depend on conformational disorder, either static or dynamic, which often cannot be fully characterized, with the consequence that the positional dispersion increases B-factors. Moreover, unless extremely high resolution diffraction data are available, crystallographic refinements require the use of restraints that reduce the differences between B-factors of atoms that are connected by a covalent bond in order ensure to the covalent bond rigidity. Furthermore, occupancies, which can be equal to one – if the atom has only one position – or minor than one – if the atom has at least two equilibrium positions – may alter the values of the B-factors, since a decrease of the occupancy implies a decrease of the B-factor. It may also happen, occasionally, that the assignment to the wrong chemical entity of an electron density peak may influence B-factors: for example, an isolated peak might be interpreted as a water molecule, with smaller B-factor, or as a cation of atomic mass larger than water, for example calcium(II), with much larger B-factor.

Although B-factors are not determined uniquely by atomic oscillations, in crystal structure refinements they are handled like oscillations and there are different levels of approximations in their modelling. In protein crystallography, B-factors are usually refined isotropically, in the sense that it is assumed that the oscillation amplitudes are identical in all directions, though this assumption is nearly never justified. Only at very high resolution, when diffraction data are sufficiently numerous, B-factors can be refined anisotropically, when three principal components of oscillation are refined independently of each other. However, also this approximation can deviate considerably from reality.

Despite these limitations, B-factors have been extensively studied and they found several interesting applications during the last decades in biological chemistry. They have been used to identify thermal motion paths, which are the dynamic and transient channels that allow molecules to enter or exit from protein internal cavities [2, 3]. They have been correlated to the rotameric state of amino acids side-chains [4], it has been possible to use them to improve protein superposition algorithms [5] and their accuracy has been investigated [6].

B-factor profiles have been used to increase the thermostability of amine transaminase [7] or lipase1 [8]. Comparisons between B-factors in mesophilic, thermophilic and psychrophilic proteins have been reported [9] and the relationship between protein thermostability and B-factors has been investigated [10].

B-factors are related to atom packing in proteins [11] and a simple algorithm has been designed to predict B-factors, based on the protein three-dimensional structure [12]. B-factor values have been predicted also on the basis of multiple picosecond molecular dynamics simulations [13]. Structure-based B-factor predictions have been reported also by Yang and colleagues [14].

Protein flexibility has been extensively investigated through B-factors [15–17]. B-factor information was used to design a sequence-based predictor of local protein flexibility [18, 19]. B-factors have also been used to distinguish crystal packing contacts from physiological protein-protein binding sites [20].

Fenwick and co-workers designed a B-factor based order parameter, which approaches its maximal value equal to one for well ordered atoms, and approaches zero for highly disordered atoms. This order parameter, for single-conformer structural models, becomes unphysical (negative) when the sum of the B-factors of two atoms approaches 8π^2^ Å^2^ (≈ 79 Å^2^) [21].

Unfortunately, it became common in recent years to deposit in the Protein Data Bank [22, 23], B-factors extremely large. While very large B-factors are usually associated with “invisible” protein moieties, the structure of which is evanescent in the electron density maps [24], atoms with incredibly high B-factors are often included in the refined three-dimensional model, despite their negligible contribution to structure factor.

This might have severe consequences on the bioinformatics use of the B-factors. For example the quantitative relationships between local stereochemistry and B-factor (and flexibility) would be misinterpreted by considering protein moieties with unreliable structures.

Traditionally, when crystallographers are unable to position atoms or residues based on electron density maps, they insert a series of warnings in PDB files. The “REMARK 465”, “REMARK 470”, “REMARK 475”, “REMARK 480” lines were used to list (i) the residues that are present in the SEQRES records but are completely absent from the coordinate section (REMARK 465); (ii) non-hydrogen atoms of standard residues which are missing from the coordinates (REMARK 470); (iii) residues modeled with zero occupancy (REMARK 475); and (iv) non-hydrogen atoms of residues modeled with zero occupancy (REMARK 480).

Obviously, this is the result of a rather subjective decision, based on the visual analysis of the electron density maps. Some crystallographers prefer to include these “invisible” protein moieties in their refinements, with the consequent inflation of the B-factors. An atom that cannot be precisely positioned tends to occupy a large portion of space to spread its electrons as much as possible.

Recently, B-factors larger than 100 Å^2^ are often deposited in the Protein Data Bank and this has three major drawbacks. First, the occupancy and the B-factor fields merge in the PDB-formatted files, with consequent problem of readability: the string “1.00 99.99” becomes “1.00100.00” when the B-factor increases from 99.99 to 100.00 Å^2^. Second, at (sinθ/λ) = 2, the scattering power of at atom with B = 5, 20 or 80 Å^2^ is only 0.54, 0.08 or 0.0001 [25] and, as a consequence, the contribution to the calculated structure factors of atoms with B-factors larger than 100 Å^2^ is absolutely negligible. *Ab absurdo*, one might introduce several atoms of Fermium wherever possible in the asymmetric unit with B-factors extremely large without consequences on the R-factor, the free-R-factor and the electron density. Third, and this is a major concern, PDB file end users, which often are not crystallographers, might be induced to use structural data that are absolutely unreliable.

It is therefore necessary to have an estimation, at least semi-quantitative, of the highest B-factor values that can be considered to be acceptable and over which atoms can be considered to be non-localizable. Apparently, only a careful analysis of the electron density map allow one to solve this problem. This is however impossible in most bioinformatics applications due to the large amount of data that must be examined.

It is impossible to define a physically meaningful threshold value, over which B-factors can be safely be used to decide that an atom must be excluded in the refined model: it would be a mere arbitrary threshold, based on some subjective assumption, for example “less than 0.05%” of the structure factor. On the contrary, it is possible to try to identify, *ab absurdo*, the B-factor of a “liquid” atom in a protein crystal. In practice, in this article, I analyze the relationship between the average B-factors of protein crystal structures and the percentages of crystal volume occupied by solvent, which can be estimated through the Matthews volume [26]. In this way, it is possible to extrapolate the B-factor expected when all the crystal volume is occupied by liquid solvent (*B_max*). Based on the analysis of a large and well selected set of protein crystal structures, it can be predicted that at very high resolution (better than 1.5 Å), *B_max* is close to 25 Å^2^, which means that the average B-factor value should not be larger than 25 Å^2^ at that resolution, while larger values are observed at lower resolution. At very low resolution (worse than 3.3 Å), *B_max* grows up to 80 Å^2^, which means, again, that the average B-factor value should not be larger than 80 Å^2^ at that resolution.

## Methods

Protein crystal structures were downloaded from the Protein Data Bank [22, 23] according to the following criteria: only X-ray crystal structures deposited together with their experimental data were considered. Moreover, structures (i) with R-factor and free-R-factor > 0.3, (ii) with data collection temperature < 95 K or > 105 K, or (iii) containing nucleic acids were discarded. The selection of the 95–105 K temperature range is due to the fact that nearly all protein crystal structures present in the Protein Data Bank have been determined at 100 K and to the fact that atomic oscillation amplitudes depend on temperature. Redundancy was reduced to 30% sequence identity. This threshold value was selected because is the lowest and therefore the more severe threshold value available in the “Advanced Search Interface” page of the Protein Data Bank (www.rcsb.org). It is however important to observe that other criteria, slightly more or less stringent, would produce similar results (though not identical), given the extreme-value distribution of pairwise protein sequence similarity scores [27]. Eight data sets were built, according to different resolution ranges and only the structures deposited in the Protein Data Bank before 2008 were considered for reasons described and explained in Results and Discussions below (see Table 1 and Additional file 1: Table S1 in the Supplementary material). Table 1 also reports, for each resolution range, the percentages of structures in the following categories: (i) with large B-factors and with missing residues, (ii) without large B-factors and without missing residues, (iii) with large B-factors and without missing residues, and (iv) without large B-factors and with missing residues.

**Table 1:** Number of protein crystal structures in the different datasets examined in the present paper

Resolution range (Å)	Number of structures	With B > 100 Å^2^ With missing residues	Without B > 100 Å^2^ Without missing residues	With B > 100 Å^2^ Without missing residues	Without B > 100 Å^2^ With missing residues
0.0–1.5	863	4%	24%	2%	70%
1.5–1.8	1853	5%	18%	1%	76%
1.8–2.1	2505	8%	14%	1%	77%
2.1–2.4	1950	18%	11%	2%	69%
2.4–2.7	1422	31%	9%	4%	56%
2.7–3.0	916	44%	6%	6%	44%
3.0–3.3	338	53%	6%	7%	34%
3.3–4.0	76	55%	5%	12%	28%

Protein atoms were divided into 24 classes according to Li and Nussinov [28]: atom type 1 (Any main-chain CA atom), atom type 2 (Any main-chain carbonyl C atom), atom type 3 (CB of Ile, Thr and Val; CG of Leu), atom type 4 (CG of Arg, Gln, Lys, Met and Pro; CG1 of Ile; CD of Lys and Pro), atom type 5 (CB of Arg, Asn, Asp, Cys, Gln, Glu, His, Leu, Lys Met, Phe, Pro, Ser, Trp and Tyr), atom type 6 (CD of Arg; CG of Glu; CE of Lys), atom type 7 (CB of Ala; CG1 of Val; CG2 of Ile, Thr, and Val; CD1 of Ile and Leu; CD2 of Leu; CE of Met), atom type 8 (CD1 of Phe, Trp,and Tyr; CD2 of Phe and Tyr; CE1 of Phe and Tyr; CE2 of Phe and Tyr; CZ of Phe; CE3, CZ2, CZ3, and CH2 of Trp), atom type 9 (CZ of Arg and Tyr; CG of His, Phe, Trp, and Tyr; CD2 of Trp; CE2 of Trp), atom type 10 (CD2 and CE1 of His), atom type 11 (CG of Asn; CD of Gln), atom type 12 (CG of Asp; CD of Glu), atom type 13 (SG of Cys), atom type 14 (SD of Met), atom type 15 (Any main-chain N atom), atom type 16 (NE1 of Trp), atom type 17 (ND1 and NE2 of His), atom type 18 (ND2 of Asn; NE2 of Gln), atom type 19 (NE, NH1, and NH2 of Arg), atom type 20 (NZ of Lys), atom type 21 (Any main-chain O atom), atom type 22 (OD1 of Asn; OE1 of Gln), atom type 23 (OD1 and OD2 of Asp; OE1 and OE2 of Glu), atom type 24 (OG of Ser; OG1 of Thr; OH of Tyr). Despite the relative data abundance, I did not want to consider each individual atom type since some of the atoms, for example the main-chain carbonyl carbon atom, are nearly identical in each residue and since a better statistics are expected by grouping similar atoms in a single and larger cluster, like for example the side-chains methyl carbon atoms of alanine, isoleucine, valine and leucine.

Percentages of crystal volume occupied by solvent (*pcVol*) were computed with the *rwcontents* program of the CCP4 software suite [29], with default parameters. Briefly, *pcVol* is computed as

3 $$ 100\left(1-\frac{Total\ mass\ of\ protein\ in\ unit\ cell}{Protein\ density\times Unit\ cell\ volume}\right) $$

by assuming a protein density of 1.34 g/cm^3^.

## Results

It is possible to compute the average B-factor (*B*) in a protein crystal structure and the percentage of volume occupied by solvent in the crystal (*pcVol*). By plotting *B* versus *pcVol*, it is then possible to extrapolate, through linear regression, the value of *B* (*B_max*) at *pcVol* = 100%, when all the atoms contained in the crystal are, *ab absurdo*, in the liquid state.

Obviously, it is mandatory to remember that this is an extrapolation and that, like any extrapolation, it might be erroneous, since no information is (and can be) available for *pcVol* close to 100%: by definition, a crystal cannot contain only amorphous material.

It is mandatory to use a set of protein crystal structures with a minimal fraction of atoms, the B-factors of which have been allowed to inflate – the common practice nowadays. For this reason, the entire PDB was analyzed and files were classified, based on the presence of atoms with B-factors larger than 100 Å^2^ and on the presence of missing atoms or residues (listed in the REMARK 465, 470, 475 or 480 lines). Therefore, it is possible to find four types of files: (i) those with large B-factors and with missing residues; (ii) those without large B-factors and without missing residues; (iii) those with large B-factors and without missing residues; and (iv) those without large B-factors and with missing residues.

**Fig. 1:**
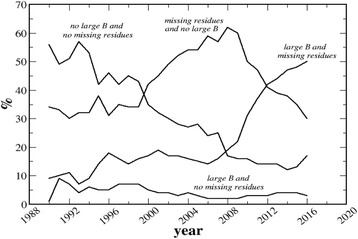
PDB entries with missing residues and large B-factors. Frequency with which different types of files have been deposited into the PDB during the last decades

As it can be seen in Fig. 1, the fraction of files without large B-factors and without missing residues is constantly decreasing with time. Analogously, the fraction of files with large B-factors and without missing residues is decreasing with time, though with a very modest slope. On the contrary, the fraction of files without large B-factors and with missing residues increases until 2008 and decreases more recently; and the fraction of files with large B-factors and with missing residues slightly increases up to 2007–2008 and increases much more markedly after 2008.

It clearly appears that the tendency to allow B-factors to reach extremely high values became common only during the last decade. For this reason, only protein crystal structures deposited in the Protein Data Bank prior 2008 were used to analyze the relationships between B-factors and *pcVol*, assuming that these data are little affected by the modern tendency to allow B-factor inflation in the absence of interpretable electron density maps.

**Fig. 2:**
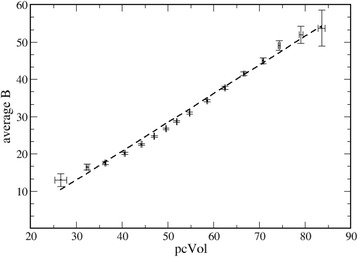
B-factors and *pcVol*. Relationship between B-factors and *pcVol* (independently of crystallographic resolution)

The relationship between B-factors and *pcVol* is shown in Fig. 2, where all protein crystal structures were considered, independently of their resolution. Average B-factors of protein atoms tend to increase if the percentage of crystal volume occupied by solvent increases (Fig. 2). On the one hand, this is expected since protein atoms can be more mobile, both in terms of oscillations around the equilibrium positions and conformational disorder, in crystals where the content of water is larger. On the other hand, it is necessary to observe that the relationship of Fig. 2 might be the consequence of other phenomena.

**Fig. 3:**
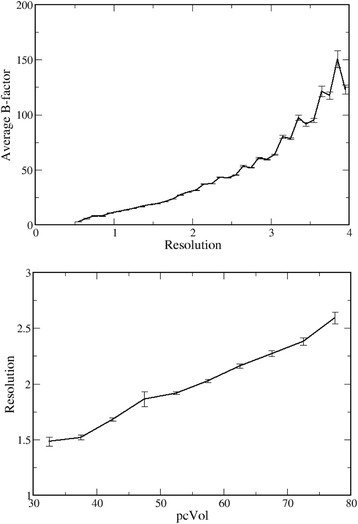
B-factors, resolution and *pcVol*. Dependence of B-factors on resolution and dependence of resolution on *pcVol* indicate that the relationship between B-factors and *pcVol* (see Fig. 2) must be examined in datasets of structures that have similar resolution

For example, it is possible to observe that crystallographic resolution decreases if *pcVol* increases (Fig. 3) and that B-factors increases if resolution decreases (Fig. 3). Consequently, it is obvious that B-factors increase if *pcVol* increases and the dependence of B-factors on *pcVol* must be analyzed for sets of protein structures refined at similar resolution.

I observe here that the dependence of the B-factor on resolution is absolutely obvious. In fact the atomic form factor (*f*_*B*_) depends on the B-factor according to

4 $$ {f}_B=f\bullet {e}^{-B{\left(\frac{sin\vartheta}{\lambda}\right)}^2} $$

which can be rewritten as

5 $$ B=-4\left(\mathit{\ln}\frac{f_B}{f}\right){resolution}^2 $$

since

6 $$ resolution=\frac{\lambda }{2\bullet sin\vartheta} $$

**Fig. 4:**
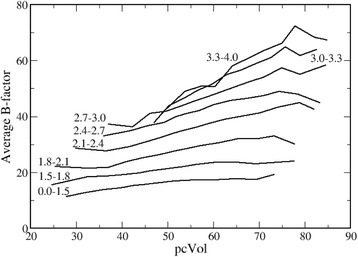
B-factors-*pcVol* versus resolution. Relationship between B-factors and *pcVol* at various resolution ranges, indicated close to each curve

The relationships between average protein B-factor and *pcVol* within various resolution ranges are shown in Fig. 4, where the bars associated with the estimated errors are omitted for clarity.

## Discussion

The correlation coefficients between B-factors and *pcVol*, although always positive, are systematically quite small. With an average value of 0.282 (standard error 0.006), they are nearly always smaller than 0.5 for the 24 atom types listed in the Methods. This depends on the considerable scatter of the average B-factors within each resolution range, which is not reported in Fig. 4 for clarity. Usually, this scatter can be reduced by using standardized B-factors (*BN*), defined as

7 $$ BN=\frac{B-{B}_{av}}{B_s} $$

where *B*_*av*_ is the average B-factor of a protein crystal structure and *B*_*s*_ is its variance. However, this is not possible here since the average *BN* is equal to zero by definition and, as a consequence, it is invariant and cannot depend on any other variable. When one analyzes a specific type of protein atoms, for example the backbone carbonylic carbon atoms, its average *BN* value can be different from zero. However, individual average *BN* values are nearly independent on resolution and *pcVol*, since the standardization is performed within a single protein crystal structure – this is actually the reason why this standardization is performed.

Apparently, B-factors tend to increase with both *pcVol* and resolution (Fig. 4).

The B-factors expected when *pcVol* tends to 100% (*B_max*) are shown in Table 2 for all 24 atom types (the Pearson correlation coefficients of the linear relationships between average *B* and *pcVol* are relatively small – they range between 0.23 and 0.51 – given the intrinsic variability of the B-factors; however, the slopes are statistically larger than zero for all atom types). *B_max* tend to increase if resolution decreases. In the highest resolution range (0.0–1.5 Å) and when all the atom types are considered together, the average B-factor at *pcVol* = 100% is only 25 Å^2^, while it is 80 Å^2^, in the lowest resolution range (3.3–4.0 Å).

**Table 2:** Values of the B-factors (Å^2^) expected when the percentage of solvent tends to 100% (*B_max*; confidence intervals in parentheses)

Atom type	Resolution range (Å)							
	0.0–1.5	1.5–1.8	1.8–2.1	2.1–2.4	2.4–2.7	2.7–3.0	3.0–3.3	3.3–4.0
Any	25(2)	31(2)	43(2)	55(3)	61(3)	70(5)	83(9)	80(19)
1	23(2)	30(2)	41(2)	53(3)	59(3)	68(5)	80(9)	77(19)
2	23(2)	30(2)	42(2)	54(3)	60(3)	68(5)	80(9)	77(19)
3	23(2)	30(2)	41(2)	52(3)	57(3)	64(5)	74(9)	71(19)
4	26(2)	33(2)	44(2)	56(3)	62(3)	70(5)	83(9)	84(20)
5	24(2)	31(2)	42(2)	54(3)	61(3)	70(5)	84(9)	84(20)
6	33(3)	39(2)	51(2)	64(3)	70(4)	80(5)	98(11)	102(22)
7	24(2)	31(2)	41(2)	51(3)	56(3)	63(5)	76(9)	72(19)
8	23(2)	30(2)	40(2)	51(3)	56(3)	64(5)	79(9)	72(19)
9	25(3)	32(2)	43(2)	55(3)	62(4)	70(5)	84(9)	81(21)
10	26(4)	32(3)	44(3)	56(4)	61(4)	72(6)	92(11)	90(23)
11	29(3)	35(2)	48(2)	59(3)	67(4)	79(5)	93(10)	94(21)
12	32(3)	39(3)	52(3)	65(3)	73(4)	86(6)	102(11)	106(22)
13	22(4)	25(3)	36(3)	45(4)	51(5)	64(7)	74(12)	57(25)
14	27(5)	30(4)	37(4)	44(5)	57(6)	65(8)	83(13)	76(22)
15	23(2)	30(2)	41(2)	53(3)	59(3)	68(5)	80(9)	76(19)
16	20(3)	29(3)	37(3)	43(4)	47(5)	56(7)	68(12)	63(22)
17	27(4)	32(3)	44(3)	56(4)	62(4)	72(6)	91(11)	90(24)
18	31(3)	36(2)	48(2)	59(3)	66(4)	79(5)	95(10)	94(21)
19	35(4)	38(3)	51(3)	62(3)	70(4)	80(6)	97(10)	100(24)
20	40(4)	43(3)	57(3)	69(3)	74(4)	80(5)	93(11)	98(22)
21	24(2)	30(2)	41(2)	54(3)	60(3)	69(5)	81(9)	77(19)
22	31(3)	37(2)	50(2)	61(3)	68(4)	80(5)	95(10)	95(21)
23	35(3)	41(3)	54(3)	67(3)	74(4)	87(6)	103(11)	107(22)
24	26(2)	32(2)	44(2)	56(3)	62(4)	71(5)	84(9)	85(19)

The 95% central coincidence intervals (*CI*) of the *B_max* values are also reported in Table 2. They were defined as [30]:

8 $$ CI=1.960\bullet S\bullet \sqrt{\frac{1}{n}+\frac{{\left(100-{pcVol}_{ave}\right)}^2}{T}} $$

where *n* is the number of points, *pcVol*_*ave*_ is the average *pcVol* value and

9 $$ S=\sqrt{\frac{\sum {\left({B}_{obs}-{B}_{calc}\right)}^2}{n-2}} $$

where *B*_*obs*_ is the observed value of the B-factor, *B*_*calc*_ is the computed value of the B-factors (calculated after linear regression of the relationship between average *B* and *pcVol*; see Fig. 2) and

10 $$ T=\sum {\left( pcVol-{pcVol}_{ave}\right)}^2 $$

*CI* values are much smaller at higher resolution. When all the atoms are considered together, *CI*s are equal to 2 Å^2^ up to 2.1 Å resolution; then they increase slowly when resolution decreases down to 3.3 Å when they are equal to 9 Å^2^; only in the lowest resolution range (3.3–4.0 Å) *CI*s are definitely vary large (nearly 20 Å^2^).

*B_max* values are smaller for main-chain atoms than for side-chain atoms. For example, they range from 23 to 24 Å^2^ in the 0.0–1.5 Å resolution range, with an average value of 23 Å^2^, while the analogous values range from 20 to 40 Å^2^ for side-chain atoms, with an average value of 28 Å^2^. In general, main-chain *B_max* values are about 5 Å^2^ smaller that side-chain *B_max* values.

*B_max* values tend to increase in going from sulfur, to carbon, to nitrogen and to oxygen atoms. For example, in the 0.0–1.5 Å resolution range, they are equal, on average, to 24 Å^2^ for sulfur atoms, to 26 Å^2^ for carbon atoms, and to 29 Å^2^ for nitrogen and oxygen atoms. It must however be observed that these differences are often statistically insignificant, given the quite wide variety of atom types per each chemical element, with the partial exclusion of sulfur.

Minimal *B_max* values are observed for the side-chain sulfur atom of cysteine (atom type 13) or the Nε1 nitrogen atom of tryptophan (atom type 16). Interestingly, the side-chain sulfur atoms of methionine (atom type 14), which is more distant from the backbone than the side-chain sulfur atom of cysteine, is also associated with larger *B_max* values. For example, in the 0.0–1.5 Å resolution range, it is equal to 27 Å^2^, while that of cysteine is equal to 22 Å^2^.

Maximal *B_max* values are observed for the side-chain nitrogen atom of lysine (atom type 20) and for the side-chain carboxylic oxygen atoms of aspartate and glutamate (atom type 23), which are at the end of long side-chains and are also, in general, largely exposed to the solvent. Interestingly, the side-chain nitrogen atoms of arginine have, in general, *B_max* values lower than the side-chain nitrogen atom of lysine and this might depend on the fact that the guanidinium moiety of arginine is larger and may for more hydrogen-bonds than the ammonium moiety of lysine.

**Fig. 5:**
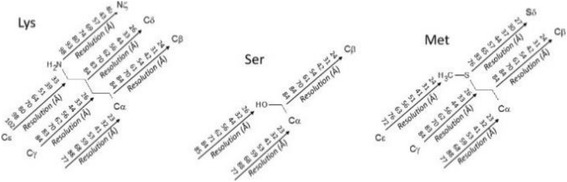
*B-max* examples. *B_max* values (Å^2^), at various resolution ranges, for the side chain atoms of lysine, serine, and methionine

Figure 5 depicts the *B_max* values for a charged residue (lysine), a polar residue (serine), and an apolar residue (methionine). It clearly appears that *B_max* values tend to increases if the distance from the backbone increases. Lysine, which has the longest side-chain that ends with a cationic group, has, as expected, the largest *B_max* values at its extremity.

*B_max* values were estimated from the analysis of protein crystal structures deposited until 2008 in the Protein Data Bank, when the tendency to allow B-factors to inflate arbitrarily was minimal. The analysis of a set of 2783 protein crystal structures deposited after 2014, assembled with the same criteria described in Methods, is summarized in Table 3. Structures deposited after 2008 and before 2014 were ignored, since this is a period of transition, when the tendency to allow B-factors to inflate to large values progressively took place. While at high resolution only a small fraction of the structures have an average B-factor larger than *B_max*, at low resolution a large fraction of the structures have an average B-factor larger than *B_max*. This depends on the fact that at high resolution only few atoms are invisible in the electron density maps and, as a consequence, few atoms have unreasonably large B-factors. On the contrary, at low resolution, several atoms are invisible in the electron density maps and have, as a consequence, extremely large B-factors. From the perspective of structural bioinformatics and of the users of the Protein Data Bank information, this means that nearly one half of the crystal structures refined at low resolution should be considered with extreme caution, since many moieties of them are not experimentally determined, but just modeled computationally. It is also interesting to observe that even at very high resolution about 15% of the protein crystal structures are likely to be incomplete, given their large average B-factor.

**Table 3:** Percentage of protein crystal structures deposited after 2014 in the Protein Data Bank that have and average B-factor larger than *B_max*

Resolution (Å)	Percentage (%)
0.0–1.5	5
1.5–1.8	17
1.8–2.1	19
2.1–2.4	30
2.4–2.7	34
2.7–3.0	37
3.0–3.3	43
3.3–4.0	76

## Conclusions

Based on well controlled data sets, it is possible to estimate the maximal B-factor average values that are compatible with the crystalline solid state (*B_max*).

*B_max* value increase, as expected, if resolution decreases. At very high resolution, better than 1.5 Å, *B_max* is equal to 25 Å^2^ and it increases to 31, 43, 55, 61, 70, 83, and 80 Å^2^ in the resolution ranges 1.5–1.8, 1.8–2.1, 2.1–2.4, 2.4–2.7, 2.7–3.0, 3.3–3.3 and > 3.5 Å.

Different *B_max* values are observed for different types of atoms. They are slightly larger for side-chain atoms than for main-chain atoms and for nitrogen and oxygen atoms than for carbon atoms or sulfur atoms. Highest *B_max* values are observed for atoms that are systematically solvent exposed and lowest *B*_*max*_ values are observed for carbon atoms involved in many covalent bonds with other non-hydrogen atoms of for sulfur atoms.

It is mandatory to remember the two main limitations of this approach. On the one hand, the *B_max* values are extrapolated at *pcVol* = 100%, while observed *pcVol* are always smaller than 80%, and, consequently, *B_max* values might be inaccurate. On the other hand, only a detailed analysis of the electron density map can allow one to decide if the atoms are “visible” despite their uncommonly large B-factors.

However, in large scale bioinformatics studies it is necessary to have an automatic criterion and *B_max* can be a reasonable threshold to identify crystal structures, which can be considered to be modelled, at least in part, rather than experimental. In a very preliminary and perhaps simplistic way, it is possible to suggest to use *B_max* in a semi-quantitative way. If a protein crystal structure has an average B-factor larger than the *B_max* corresponding to its crystallographic resolution, some of the protein atoms are likely to have been refined with too large B-factors to be considerable as “visible”. This would be certainly true if the large majority of the B-factors seem acceptable and only a small minority of the atoms have extremely large B-factors. Different is the case when many atoms, perhaps nearly all the atoms, have B-factors large and close to, if not larger than, the *B_max* value.

In a crystal structure with average B-factor larger than *B_max*, the atoms associated with the largest B-factors might be eliminated, until the average B-factor of the remaining atoms falls under the *B-max* threshold.

Although further analyses and alternative strategies need to be designed and tested, it is irrational to deposit atoms with, sometime, astronomically large B-factors in the Protein Data Bank without an explicit declaration about the invisibility of these atoms in the electron density map. Otherwise, over-interpretations of the data are possible and scientifically deleterious. A typical example is the use of structural models in computational docking simulations, where minor deformations of the protein surface can have major consequences on the simulation results.

## Additional file

Additional file 1: **Table S1.** List of the identification codes of the PDB files of the datasets examined in the present paper. (DOCX 37 kb)

## Abbreviations

*B-max*: B-factor upper limit (corresponding to *pcVol* = 100%)
*pcVol*: Percentage of crystal volume occupied by solvent.

## References

[1] Kuzmanic A, Pannu NS, Zagrovic B (2014). X-ray refinement significantly underestimates the level of microscopic heterogeneity in biomolecular crystals. Nat Commun.

[2] Carugo O, Argos P (1998). Accessibility to internal cavities and ligand binding sites monitored by protein crystallographic thermal factors. Proteins.

[3] Luedemann S, Carugo O, Wade RC (1997). Substrate access to cytochrome P450can: a comparison of a thermal motion pathway analysis with molecular dynamics simulation data. J Mol Model.

[4] Carugo O, Argos P (1997). Correlation between side chain mobility and conformation in protein structures. Protein Eng.

[5] Carugo O, Eisenhaber F (1997). Probabilistic evaluation of similarity between pairs of three-dimensional protein structures utilizing temperature factors. J Appl Crystallogr.

[6] Carugo O, Argos P (1999). Reliability of atomic displacement parameters in protein crystal structures. Acta Crystallogr D Biol Crystallogr.

[7] Huang J, Xie DF, Feng Y (2017). Engineering thermostable (R)-selective amine transaminase from aspergillus terreus through in silico design employing B-factor and folding free energy calculations. Biochem Biophys Res Commun.

[8] Zhang XF, Yang GY, Zhang Y, Xie Y, Withers SG, Feng Y (2016). A general and efficient strategy for generating the stable enzymes. Sci Rep.

[9] Siglioccolo A, Gerace R, Pascarella S (2010). “Cold spots” in protein cold adaptation: insights from normalized atomic displacement parameters (B-factors). Biophys Chem.

[10] Parthasarathy S, Murthy MR (2000). Protein thermal stability: insights from atomic displacement parameters (B values). Protein Eng.

[11] Yin H, Li YZ, Li ML (2011). On the relation between residue flexibility and residue interactions in proteins. Protein Pept Lett.

[12] Weiss MS (2007). On the interrelationship between atomic displacement parameters (ADPs) and coordinates in protein structures. Acta Crystallogr.

[13] Pang YP (2016). Use of multiple picosecond high-mass molecular dynamics simulations to predict crystallographic B-factors of folded globular proteins. Heliyon.

[14] Yang J, Wang Y, Zhang Y (2016). ResQ: an approach to unified estimation of B-factor and residue-specific error in protein structure prediction. J Mol Biol.

[15] Vihinen M, Torkkila E, Riikonen P (1994). Accuracy of protein flexibility predictions. Proteins.

[16] Parthasarathy S, Murthy MRN (1997). Analysis of temperature factor distribution in high-resolution protein structures. Protein Sci.

[17] Parthasarathy S, Murthy MRN (1999). On the correlation between the main-chain and side-chain atomic displacement parameters (B values) in high-resolution protein structures. Acta Crystallogr.

[18] de Brevern AG, Bornot A, Craveur P, Etchebest C, Gelly JC (2012). PredyFlexy: flexibility and local structure prediction from sequence. Nucleic Acids Res.

[19] Smith DK, Radivojac P, Obradovic Z, Dunker AK, Zhu G (2003). Improved amino acid flexibility parameters. Protein Sci.

[20] Liu Q, Li Z, Li J (2014). Use B-factor related features for accurate classification between protein binding interfaces and crystal packing contacts. BMC Bioinformatics.

[21] Fenwick RB, van den Bedem H, Fraser JS, Wright PE (2014). Integrated description of protein dynamics from room-temperature X-ray crystallography and NMR. Proc Natl Acad Sci U S A.

[22] Berman HM, Westbrook J, Feng Z, Gilliland G, Bhat TN, Weissig H, Shindyalov IN, Bourne PE (2000). The Protein Data Bank. Nucleic Acids Res.

[23] Bernstein FC, Koetzle TF, Williams GJB, Meyer EFJ, Brice MD, Rodgers JR, Kennard O, Shimanouchi T, Tasumi M (1977). The protein data Bank: a computer-based archival file for macromolecular structures. J Mol Biol.

[24] Djinovic Carugo K, Carugo O (2015). Missing strings of residues in protein crystal structures. Intrinsically Disordered Proteins.

[25] Cruickshank DWJ (1999). Remarks about protein structure precision. Acta Cryst.

[26] Matthews BW (1968). Solvent content of protein crystals. J Mol Biol.

[27] Levitt M, Gerstein M (1998). A unified statistical framework for sequence comparison and structure comparison. Proc Natl Acad Sci U S A.

[28] Li AJ, Nussinov R (1998). A set of van der Waals and coulombic radii of protein atoms for molecular and solvent-accessible surface calculation, packing evaluation, and docking. Proteins.

[29] Collaborative Computational Project N (1994). The CCP4 suite: programs for protein crystallography. Acta Crystallogr D Biol Crystallogr.

[30] Dowdy S, Wearden S (1991). Statistics for research.

