# Supplementary material for: How large B-factors can be in protein crystal structures
Source: BMC Bioinformatics. 2018 Feb 23;19:61. doi: 10.1186/s12859-018-2083-8 (PMC5824579; doi:10.1186/s12859-018-2083-8)
Supplement: Supplementary file 1 — Table S1. List of the identification codes of the PDB files of the datasets examined in the present paper. (DOCX 37 kb) [file 12859_2018_2083_MOESM1_ESM.docx]

How large B-factors can be in protein crystal structures

**Oliviero Carugo**

Department of Chemistry, University of Pavia, viale Taramelli 12, I-27100 Pavia, Italy

Department of Structural and Computational Biolo gy, University of Vienna, Campus Vienna Biocenter 5, A-1030 Vienna, Austria

# Supplementary material

**Table S1**. List of the identification codes of the PDB files of the datasets examined in the present paper.

Resolution 0.0-1.5

863 files

1a4i 1atg 1b16 1bgf 1bqc 1bte 1c0q 1c9o 1cip 1cru 1cs1 1cxq 1czq 1d4o 1d4t 1dci 1di6 1dj0 1djr 1ds1 1dzk 1e19 1e30 1e5k 1e6u 1e7l 1eaj 1eaq 1eb6 1ejg 1elk 1ep0 1es5 1es9 1et1 1eu1 1euw 1ew0 1ew4 1ezg 1f0i 1f1e 1f46 1f7d 1f7l 1f86 1f9i 1f9v 1fd3 1fg7 1fgy 1fj2 1fl0 1fm0 1fo8 1fp2 1fsg 1fx2 1fy2 1g1t 1g2r 1g3p 1g6c 1g7a 1ga6 1gk7 1gk9 1gkp 1gmu 1gmx 1gnl 1gp0 1gpp 1gs5 1gut 1gwu 1gxm 1gxu 1h05 1h12 1h32 1h41 1h4a 1h4x 1hbn 1hnj 1ht6 1hw1 1hx0 1hyo 1hz4 1hzt 1i0r 1i12 1i1j 1i1w 1i24 1i2t 1i4u 1i52 1i71 1i88 1i8o 1ijy 1ikp 1inl 1io0 1iom 1iq6 1iqz 1irq 1is9 1isp 1itx 1j0p 1j2r 1j3w 1j77 1j8q 1j97 1j98 1jcd 1jek 1jf8 1jfb 1ji7 1jig 1jl1 1jm1 1jnd 1jo0 1jr8 1jx6 1jy2 1jyk 1jz8 1k0m 1k20 1k2a 1k3i 1k3y 1k49 1k5n 1k6u 1k7j 1k7k 1k8u 1ka1 1kdg 1kg2 1kgd 1kgs 1kjq 1kko 1kll 1kmt 1kmv 1knm 1kq3 1kq6 1kqr 1kr4 1kr7 1krh 1kt6 1ktn 1kv7 1kw3 1kyc 1l3k 1l6r 1l7a 1l7l 1l9l 1lc0 1lc5 1llf 1lm4 1lq9 1lqt 1lr7 1ls1 1ltz 1lu0 1lug 1lzj 1lzl 1m0k 1m15 1m1f 1m1n 1m1q 1m22 1m2d 1m40 1m55 1m70 1m7g 1m9z 1me3 1mg4 1mj5 1mkk 1mqd 1mqo 1mtp 1muw 1mwq 1mxr 1my7 1n13 1n3l 1n4w 1n55 1n7o 1n7s 1n8k 1n8v 1na5 1nf9 1ng6 1nh0 1nkg 1nlq 1nn5 1nnf 1nqj 1ntv 1nuy 1nwa 1nww 1nyc 1nyk 1nyt 1nz0 1nzi 1nzj 1o06 1o2d 1o4y 1o6v 1o8b 1o8x 1o9g 1o9i 1o9r 1oaa 1oai 1obo 1oc7 1ocy 1od3 1od6 1odm 1odz 1of8 1ofz 1oh0 1oh4 1oi0 1oi6 1oi7 1ojj 1ojr 1oki 1olr 1omr 1ooh 1oqv 1os6 1ox0 1oxx 1oyg 1p1m 1p3c 1p5f 1p6o 1pa7 1pfb 1pin 1pkh 1pko 1pqh 1pvm 1pwa 1pwb 1pwm 1pyz 1pz7 1q0n 1q0r 1q1a 1q35 1q5y 1q6o 1q6z 1q7l 1qau 1qb7 1qfm 1qgq 1qh5 1qks 1qlw 1qop 1qre 1qs1 1qtw 1qv1 1qw2 1qw9 1qwo 1qwy 1qx2 1qyz 1qz0 1r29 1r5l 1r5y 1r6j 1r6x 1r7j 1ra0 1rg8 1rh9 1rju 1rk6 1rku 1rtq 1rtt 1rut 1rw1 1rwh 1ryo 1ryq 1s0p 1s2o 1s67 1s8n 1s9u 1sen 1sfs 1sg0 1sg4 1sh8 1shu 1sj1 1sjw 1sjy 1sn9 1so7 1sqe 1sqs 1ssx 1su8 1svf 1sx7 1t1e 1t2d 1t2h 1t3y 1t5b 1t6f 1t6u 1t7h 1t7r 1t8k 1tg0 1tgr 1thf 1tjy 1tke 1tkj 1tp6 1tqg 1tqw 1tt8 1tu9 1tua 1tuk 1tzp 1tzv 1u07 1u2h 1u4g 1u7g 1u7i 1uai 1uas 1ucd 1ucr 1ufy 1ug6 1ui0 1ukf 1uku 1ulr 1uo9 1uow 1uq5 1urr 1urs 1us5 1usc 1use 1usm 1uuq 1uuy 1uv4 1uwc 1uww 1uxz 1uyl 1v05 1v2x 1v30 1v37 1v3w 1v4f 1v4p 1v5v 1v6p 1v6s 1v70 1v7h 1v7r 1v8h 1v9y 1vbw 1vc3 1vd6 1vdw 1ve1 1ve4 1vef 1vf8 1vfy 1vk1 1vkk 1vl7 1vl9 1vlb 1vly 1vm9 1vmg 1vmh 1vp8 1vqs 1vyi 1w0n 1w1h 1w5q 1w5r 1w7c 1w8u 1wb4 1wbe 1wc2 1wck 1wcw 1wdd 1wkq 1wkr 1wl8 1wlu 1wmh 1wn2 1wpa 1wpn 1wri 1wrm 1wrv 1wui 1wvf 1wvh 1wxc 1wyx 1wzd 1x1z 1x2i 1x46 1x54 1x6i 1x6z 1x82 1x8q 1x91 1x99 1x9d 1x9i 1xaw 1xbi 1xdn 1xg0 1xgk 1xmt 1xod 1xqo 1xrk 1xt5 1xub 1y0p 1y37 1y5h 1y6x 1y93 1y9z 1ybi 1yd0 1ye8 1yfq 1yge 1yjo 1yk4 1ymt 1yn9 1yph 1ypq 1yqs 1ys1 1yu5 1yzm 1z0n 1z0w 1z1s 1z21 1z2n 1z2u 1z3e 1z3x 1z67 1z6m 1z6n 1z70 1z72 1zbf 1zce 1zd8 1zdy 1zeq 1zgk 1zgx 1zhv 1zi8 1zk5 1zkp 1zl0 1zma 1zmi 1zuu 1zuy 1zv9 1zva 1zzk 2a13 2a26 2a35 2a3n 2a50 2a6y 2a6z 2a8y 2abb 2abs 2ad6 2agk 2agy 2ahn 2aib 2akf 2akz 2aml 2anx 2arc 2asc 2au7 2axw 2ayw 2b02 2b06 2b0p 2b5h 2b69 2b82 2b97 2bay 2bbr 2bcm 2bfd 2bfq 2bhu 2bjd 2bk9 2bkm 2bln 2bmo 2bmw 2bog 2bqz 2brf 2brj 2bry 2bsy 2bu3 2bw4 2bwr 2bzv 2c1i 2c1v 2c2u 2c3n 2c4b 2c5a 2c60 2c61 2c71 2c78 2c9v 2cb8 2cbz 2cdc 2ce0 2ce2 2cf7 2cfe 2cg7 2chh 2cia 2cih 2cit 2ciw 2cjl 2cjt 2cnq 2cov 2cs7 2cvi 2cws 2cxn 2cyg 2cyj 2cz2 2cz6 2cz8 2czq 2d5m 2d5w 2d8d 2dcf 2ddr 2ddx 2dej 2dfb 2dkj 2dko 2dpf 2dpl 2dq6 2ds5 2dsk 2dsn 2dt8 2dxu 2dy0 2e3b 2e3h 2e3n 2e4t 2e5f 2e6f 2erb 2erf 2erw 2etx 2eut 2ew0 2ewh 2f01 2f22 2f46 2f5v 2f62 2f69 2f9s 2fao 2fb6 2fba 2fcj 2fcl 2fco 2fcw 2ffy 2fg1 2fgq 2fi1 2fj8 2fkk 2fla 2fma 2fnu 2fom 2fr2 2fr5 2frg 2fsq 2ft6 2ftr 2fuf 2ful 2fup 2fvv 2fvy 2fwh 2fxu 2fyf 2g1u 2g3d 2g3r 2g7o 2g7s 2g84 2g8s 2gcu 2gdg 2gf3 2ggc 2ghc 2gj3 2gke 2gkg 2gkp 2glz 2gmn 2gmw 2gqv 2gqw 2gs5 2gs8 2gso 2gu9 2gud 2gxg 2gxq 2gyp 2gyq 2gz4 2gzq 2h1v 2h3l 2h6f 2h6n 2h8e 2h8g 2hal 2hba 2hbw 2hd9 2hdo 2heu 2hew 2hhj 2hin 2hiy 2ho2 2hox 2hqx 2hqz 2hsj 2hw2 2hx5 2hxm 2hy6 2hyk 2hyv 2hzc 2hzl 2i3d 2i3f 2i49 2i4a 2i53 2i5u 2i5v 2i7d 2i8t 2ia4 2ia7 2iay 2ibd 2ibl 2ibn 2ic6 2icc 2ij2 2ik9 2imf 2imj 2imq 2ims 2ip6 2iqy 2ivy 2ixm 2ixt 2iyv 2izr 2izx 2j2j 2j32 2j5y 2j6b 2j6l 2j8b 2j8m 2j8w 2j9c 2j9o 2j9w 2jae 2jc5 2jc9 2nls 2nlv 2nn5 2nn8 2nqw 2nr7 2nrl 2nrr 2nrt 2nsz 2nwd 2nwf 2nxb 2nxw 2nyb 2nzl 2o02 2o0b 2o1q 2o23 2o2x 2o31 2o3s 2o5g 2o6n 2o6x 2o6y 2o7a 2o7i 2o7r 2o90 2o9c 2o9u 2oa2 2oa9 2ob3 2oc3 2odk 2src 2tnf 3ezm 7a3h

Resolution 1.5-1.8

1853 files

1a12 1a3c 1a3h 1a4i 1a54 1a62 1a8o 1atz 1b20 1b59 1b6a 1b7v 1b9m 1bd0 1bn7 1bqc 1bte 1btk 1bu3 1bu8 1bup 1bx4 1c3c 1c3p 1c44 1c48 1c7s 1ccz 1cip 1cjc 1cjw 1cqm 1cru 1cs1 1cs6 1cs8 1cxy 1cy9 1czf 1czq 1d0c 1d0q 1d2n 1d2s 1d3g 1d4a 1d5n 1dak 1dci 1deu 1df7 1dj0 1dj7 1dje 1dk8 1dki 1dl5 1dlj 1dll 1dlw 1dly 1dmg 1dnl 1dow 1dqe 1dqn 1dqs 1dus 1dwk 1dxe 1dy7 1dyp 1e19 1e2k 1e2w 1e30 1e5m 1e6b 1e6p 1e6y 1e71 1e7s 1ean 1ear 1edq 1ee2 1eex 1ek6 1el5 1elk 1elu 1elv 1elw 1eok 1ep0 1eqj 1eqt 1erz 1es2 1esj 1eyh 1eyv 1f0l 1f0y 1f1m 1f2p 1f2t 1f32 1f3u 1f46 1f5n 1f5v 1f7l 1f8m 1f9t 1fa8 1fao 1fc6 1fcq 1fd4 1fe6 1fg7 1fgt 1fgy 1fhu 1fj2 1fjj 1fl0 1fma 1fnl 1fo9 1fob 1fpo 1fpx 1fqt 1fs7 1fuk 1fvg 1fxo 1g12 1g1t 1g2a 1g2q 1g55 1g5t 1g60 1g69 1g6g 1g81 1g87 1g8e 1g8q 1gd0 1gde 1gej 1gm9 1gmi 1gmu 1gn0 1gnu 1gny 1go3 1gp6 1gq6 1gs5 1gsv 1gte 1gtk 1gtv 1gu7 1gut 1guu 1gvg 1gvj 1gw1 1gxn 1gxr 1gxy 1gy7 1gz2 1gzc 1h03 1h05 1h14 1h16 1h2e 1h32 1h41 1h4c 1h4p 1h4r 1h4y 1h6h 1h6t 1h72 1h75 1h7c 1h80 1h8u 1h99 1h9m 1ha1 1hch 1hfe 1hkh 1hm9 1hnd 1hp1 1ht6 1htw 1hw1 1hx6 1hxi 1hxk 1hxr 1hyv 1hz6 1i0r 1i0x 1i1d 1i2c 1i2h 1i4y 1i52 1i58 1i60 1i6l 1i6w 1i86 1i8f 1i9s 1i9z 1iby 1id0 1igq 1ihg 1ihj 1ihr 1iir 1ijb 1ikq 1ikt 1im5 1in4 1inl 1inn 1iom 1ioo 1iq4 1iq6 1iqc 1iqq 1irq 1iu1 1iu8 1iue 1iuj 1iuq 1iv2 1ix2 1iyn 1izc 1j02 1j1m 1j1y 1j24 1j2l 1j2w 1j30 1j31 1j3w 1j58 1j77 1j79 1j7x 1j83 1j8b 1j97 1ja1 1jak 1jay 1jb6 1jbk 1jcc 1jek 1jfu 1jfx 1jg8 1jh6 1jhd 1jhf 1jhj 1ji1 1jix 1jjf 1jke 1jks 1jkx 1jlj 1jlv 1jm0 1jmk 1jne 1jp4 1jpz 1jq0 1jqc 1jr8 1jsd 1jsr 1jtv 1juh 1juv 1jvw 1jwq 1jx6 1jxg 1jy3 1jyd 1jye 1jyh 1jyk 1jz8 1k07 1k0i 1k0r 1k1e 1k20 1k2x 1k3l 1k49 1k4n 1k4v 1k50 1k6k 1k6x 1k75 1k7i 1k7k 1k92 1k94 1k9u 1k9z 1kaf 1kcq 1kdg 1ke1 1kfw 1kg6 1kgb 1kgs 1khc 1khi 1khx 1kic 1kid 1kjr 1kk1 1kll 1km4 1kn3 1kq1 1kq3 1kqf 1kr7 1krh 1ks2 1ks9 1ktg 1kux 1kwg 1kwm 1kx9 1kyh 1kzq 1l0g 1l1l 1l2h 1l7a 1l7l 1l8b 1l8r 1l9x 1lb6 1lbv 1lc3 1lc8 1lfp 1lj8 1lj9 1lkd 1lkp 1lm5 1ln4 1lr7 1ltu 1lvm 1lw6 1lxj 1lyc 1m0u 1m0w 1m1p 1m1s 1m2a 1m2x 1m33 1m3u 1m4d 1m4j 1m65 1m6k 1m7j 1m7y 1m8a 1m8u 1m93 1mc9 1mg4 1mg5 1mgq 1mhn 1mi4 1mid 1mix 1mjh 1mk0 1mk4 1mkz 1ml4 1mlw 1moy 1mpg 1mr7 1msv 1mtp 1mug 1mv8 1mve 1mw9 1mwp 1mxg 1mxi 1mz4 1mz9 1n08 1n0w 1n1j 1n1t 1n28 1n3b 1n45 1n5q 1n6a 1n7h 1n7o 1n93 1n9e 1n9p 1na0 1na3 1na5 1nb9 1nc5 1nc7 1ne2 1ne9 1nep 1nf9 1ng2 1nh8 1njh 1nkg 1nln 1nlq 1nme 1nn5 1nnh 1nnl 1nqd 1nr0 1nrj 1nrz 1ntf 1nth 1ntv 1nty 1nu0 1nu3 1nu4 1nux 1nvm 1nwa 1nxc 1nxu 1nyt 1nzi 1nzj 1o1y 1o1z 1o26 1o3u 1o54 1o58 1o5k 1o6v 1o7e 1o7u 1o97 1o9g 1o9w 1oa1 1oa8 1oad 1oag 1ob8 1oc6 1ocy 1od6 1of4 1of8 1ofl 1ofn 1ofz 1ogq 1ohp 1oi0 1oi1 1oi2 1ojh 1ojk 1ok7 1oks 1omr 1on2 1ooe 1ooy 1opk 1oq1 1oq5 1oqj 1oru 1orv 1osy 1ov3 1ow1 1ow4 1owl 1oxs 1oyg 1oz2 1ozn 1p0h 1p0z 1p13 1p1j 1p1m 1p3c 1p3d 1p5d 1p5v 1p80 1p99 1p9a 1p9h 1pb1 1pby 1pc4 1pcf 1pe1 1pe9 1pee 1pf3 1pfv 1pg6 1pgv 1pj5 1pm4 1pn0 1ppv 1pq1 1pqm 1prz 1pv5 1pvg 1pvm 1pw9 1px5 1pxv 1pxz 1py9 1pzs 1q0g 1q0p 1q11 1q1a 1q2h 1q2u 1q3l 1q42 1q74 1q7e 1q7z 1q8d 1q91 1q9u 1qb7 1qd9 1qe3 1qf8 1qgq 1qh7 1qh8 1qho 1qhv 1qip 1qiq 1qjp 1qkr 1qmg 1qo7 1qq0 1qq5 1qqj 1qr9 1qrf 1qrx 1qs1 1qw2 1qw8 1qwd 1qwg 1qwi 1qwl 1qwo 1qwr 1qwz 1qyf 1qz0 1r0q 1r0u 1r1g 1r1t 1r2r 1r3s 1r45 1r55 1r5l 1r5m 1r62 1r6v 1r77 1r88 1r89 1r8m 1r8n 1r9h 1r9l 1r9w 1r9x 1rao 1rc9 1rcv 1rdo 1rfy 1rh9 1riy 1rjd 1rk4 1rki 1rli 1rm6 1rnj 1ro2 1ro7 1roa 1rp0 1rqp 1rre 1rrm 1ru0 1ru4 1ruw 1rwg 1rwi 1rwj 1ryb 1ryi 1ryl 1rz2 1rzh 1rzy 1s1d 1s22 1s3e 1s5a 1s5u 1s67 1s7i 1s95 1s9r 1sbx 1sc0 1sd5 1sdi 1se0 1sf9 1sfx 1sg0 1sgw 1sh8 1shu 1sj1 1smb 1smx 1sna 1sqe 1sqg 1sqs 1su2 1su6 1suu 1svd 1sw5 1sxr 1syy 1sz7 1szn 1t00 1t07 1t0t 1t1d 1t1j 1t1u 1t1v 1t2b 1t2w 1t3i 1t4b 1t56 1t6c 1t6e 1t7m 1t82 1t92 1t9p 1ta8 1tcv 1te2 1tfj 1tfz 1th7 1thz 1tj6 1tjo 1tjp 1tkg 1tmx 1tov 1tp5 1tp6 1tq5 1tqh 1tqj 1tr0 1tr9 1ts9 1tua 1tuv 1tvg 1twd 1twy 1txl 1txn 1tyj 1tzw 1u0a 1u11 1u1s 1u1v 1u55 1u5d 1u5x 1u60 1u6k 1u6r 1u7l 1u84 1u8t 1u8v 1ual 1uaq 1uar 1uas 1uch 1ud9 1ueh 1uek 1ufo 1ugn 1uhk 1ui0 1ui9 1uj2 1uj8 1ujn 1uk8 1ukk 1uln 1umg 1umj 1umk 1umz 1unp 1uo9 1uov 1urm 1urr 1us5 1usd 1usg 1ut1 1ute 1uuj 1uuq 1uv4 1uv7 1uwf 1uwl 1ux7 1uxo 1uxs 1uyz 1uza 1uzp 1v0t 1v2x 1v3h 1v3w 1v4v 1v58 1v5d 1v5v 1v6s 1v6t 1v77 1v7w 1v7z 1v8c 1v8i 1v96 1v9f 1vb6 1vc3 1vc4 1vcd 1vcl 1vdk 1ve2 1vj2 1vjo 1vjv 1vk5 1vk8 1vke 1vkf 1vki 1vkp 1vl1 1vl7 1vla 1vlp 1vm0 1vma 1vmb 1vme 1vmj 1vns 1vp6 1vpb 1vpd 1vpm 1vpr 1vpt 1vqq 1vqs 1vr8 1vr9 1vrm 1vyb 1vyi 1vzg 1w0h 1w1d 1w2f 1w2i 1w2u 1w2y 1w3o 1w41 1w4s 1w4x 1w53 1w5m 1w6g 1w6u 1w79 1w7b 1w7z 1w8k 1w8o 1w96 1w99 1w9a 1w9e 1w9i 1wa0 1wb0 1wcf 1wcu 1wcv 1wd3 1wd7 1wdv 1wdy 1weh 1wer 1whz 1wka 1wkc 1wko 1wku 1wky 1wl4 1wle 1wli 1wlz 1wmh 1wmw 1wn5 1wny 1wo8 1woj 1wol 1woq 1wov 1wp5 1wpa 1wqj 1wr8 1wrv 1ws8 1wsw 1wt6 1wta 1wtf 1wtj 1wu9 1wub 1wul 1wv3 1wvh 1wvm 1wwi 1wwr 1wwz 1wx4 1wy2 1wyc 1wz3 1wza 1wzb 1wzz 1x0c 1x0t 1x1n 1x2j 1x38 1x3k 1x46 1x55 1x6o 1x6q 1x6v 1x7d 1x7s 1x7y 1x82 1x91 1x9h 1xby 1xcr 1xdz 1xe7 1xfi 1xfk 1xfs 1xg4 1xg5 1xiy 1xk4 1xk7 1xki 1xkr 1xm3 1xm8 1xoc 1xov 1xpm 1xpp 1xq6 1xqp 1xrf 1xrk 1xro 1xs1 1xsv 1xtt 1xty 1xv5 1xvb 1xvx 1xw3 1xww 1xx1 1xy7 1xzo 1xzz 1y02 1y07 1y0h 1y0p 1y0u 1y1p 1y2m 1y2t 1y37 1y4m 1y4t 1y4w 1y5h 1y5w 1y63 1y66 1y7b 1y7r 1y7y 1y80 1y81 1y9i 1yb3 1yb6 1ybi 1ybx 1yc9 1ycd 1yd0 1ydy 1yfn 1ygt 1yi9 1yib 1yj7 1yjp 1yki 1yks 1ylh 1yll 1ylx 1ym3 1yn9 1ynb 1yo3 1yoc 1yod 1yp5 1ypy 1yqz 1yrb 1yrk 1ys2 1ysr 1yt3 1ytl 1ytq 1yu0 1yu7 1yum 1yv4 1yx1 1yxy 1yzm 1z02 1z0b 1z0p 1z0s 1z21 1z32 1z3e 1z3q 1z3x 1z4r 1z6f 1z6n 1z78 1z7a 1z96 1z9l 1z9n 1z9t 1zai 1zar 1zbf 1zd0 1zd7 1zed 1zeq 1zhs 1zhv 1zi9 1zja 1zjc 1zk7 1zke 1zki 1zko 1zkp 1zld 1zmh 1zmm 1zmp 1zn6 1znd 1zos 1zpe 1zps 1zpw 1zr6 1zrs 1zru 1zs0 1zs9 1zsw 1zsy 1zt3 1zuo 1zva 1zvb 1zvt 1zvz 1zw0 1zw6 1zxu 1zy7 1zzm 1zzw 2a0m 2a0n 2a10 2a14 2a15 2a2c 2a2k 2a32 2a35 2a4o 2a5l 2a5s 2a61 2a65 2a6b 2a6s 2a6v 2a7a 2a7b 2a7m 2a84 2a94 2a9d 2a9s 2aal 2abw 2ad6 2aef 2aen 2aeu 2aex 2ah6 2ahf 2aij 2aj6 2aj7 2amj 2aml 2ap3 2apj 2aq5 2aq9 2aqj 2ar1 2ar5 2arc 2arr 2as0 2asf 2ask 2atb 2av4 2avd 2avk 2avv 2awg 2axo 2axq 2ayd 2b02 2b0j 2b0p 2b0t 2b0v 2b18 2b1f 2b1y 2b29 2b2h 2b3f 2b3l 2b4h 2b4v 2b5a 2b5h 2b5w 2b61 2b7u 2b8i 2b8m 2b9e 2bay 2bba 2bc3 2bdr 2bem 2beq 2bez 2bf5 2bfq 2bfw 2bgi 2bh4 2bhy 2bii 2bjn 2bjq 2bju 2bjv 2bk8 2bka 2bkf 2bkl 2bkm 2bmw 2bnj 2bnm 2bo9 2bod 2bp6 2bq4 2bqz 2brj 2bs6 2bsw 2bsy 2buo 2bv2 2bv9 2bwr 2bwv 2bz1 2bzg 2bzu 2c0a 2c0g 2c0h 2c0z 2c1g 2c2f 2c2i 2c2n 2c2p 2c2t 2c31 2c3n 2c42 2c46 2c4e 2c53 2c54 2c5q 2c61 2c6q 2c77 2c79 2c81 2c8r 2c8s 2c92 2c95 2cb2 2cb9 2cbo 2cbz 2cc0 2ccm 2cd7 2cdc 2cey 2cf7 2cfe 2cfm 2cft 2cg6 2cgh 2chc 2chm 2ci7 2ci9 2cih 2cis 2ciu 2cj2 2cj4 2cjl 2cjs 2cki 2cmp 2co3 2cu3 2cvb 2cve 2cvi 2cwc 2cwl 2cwr 2cx1 2cx7 2cxa 2cxh 2cxs 2cxx 2cxy 2cyj 2cyy 2cz6 2cz8 2czl 2d1c 2d1e 2d1z 2d29 2d37 2d3y 2d4n 2d4p 2d59 2d5b 2d68 2d73 2d8e 2dbn 2dbq 2dby 2dc4 2ddf 2ddt 2de6 2dej 2dek 2df8 2dfy 2dg1 2dg5 2dga 2dge 2dho 2djx 2dkh 2dok 2dpf 2dpm 2dq6 2dqa 2dql 2dqw 2drv 2ds0 2ds5 2dsj 2dsk 2dsn 2dt4 2dtc 2dtj 2dur 2duy 2dvk 2dvm 2dvn 2dvt 2dwc 2dwu 2dxa 2dy1 2dya 2dyr 2e01 2e0p 2e11 2e12 2e1n 2e2r 2e3o 2e54 2e64 2eif 2erb 2eso 2et1 2etb 2etj 2etv 2eus 2ev1 2evb 2eve 2evr 2ewr 2ex0 2ex2 2ex4 2exr 2ext 2ez9 2f0c 2f1f 2f1k 2f1n 2f1w 2f21 2f23 2f26 2f4q 2f5g 2f5x 2f60 2f62 2f6d 2f6r 2f6u 2f9f 2f9h 2fa1 2fa5 2fae 2faf 2fao 2fbh 2fbn 2fbq 2fc3 2fck 2fct 2fd4 2fd5 2fd7 2fdv 2fe3 2fex 2ffc 2ffu 2fgr 2fh1 2fhf 2fhp 2fi9 2fj9 2fje 2fjz 2fk9 2fl4 2fmm 2fnu 2fom 2foz 2fp1 2fp7 2fpe 2fph 2fpq 2fpr 2fq4 2fqp 2fqt 2fqx 2fr2 2fsr 2fsu 2fsx 2ft0 2ft8 2ftn 2fu4 2fue 2fuj 2fuk 2ful 2fur 2fvh 2fw0 2fwe 2fwv 2fy7 2fyf 2fym 2fzv 2g0c 2g0w 2g13 2g1u 2g2c 2g30 2g3t 2g40 2g5f 2g5r 2g5x 2g5z 2g62 2g64 2g7e 2g82 2g8j 2g8s 2g9e 2ga8 2gai 2gc9 2gci 2gen 2gfe 2gfq 2ggo 2ggs 2gha 2ghs 2ght 2gi3 2gib 2giy 2gj4 2gkj 2gl5 2gmq 2gmw 2gmy 2gn0 2gnp 2gpi 2gr3 2gs5 2gs8 2gsf 2gsn 2gu2 2gu3 2guh 2gui 2guy 2gv2 2gvk 2gw4 2gwg 2gwh 2gyz 2gz4 2gzj 2h1c 2h1t 2h2z 2h30 2h5y 2h6f 2h6n 2h7j 2h7w 2h88 2h8g 2h8o 2h8x 2h98 2ha8 2hai 2haz 2hb5 2hbt 2hbv 2hc8 2hcf 2hdo 2he2 2heg 2hes 2hfk 2hfs 2hhc 2hhj 2hhp 2hi0 2hia 2hje 2hk6 2hkd 2hkv 2hl7 2hly 2hmj 2hnf 2hng 2hoq 2hor 2hpj 2hps 2hqh 2hqy 2hrg 2hsj 2htd 2htx 2hu9 2huh 2huj 2hvf 2hwm 2hwn 2hx0 2hx5 2hxi 2hxt 2hxv 2hxw 2hy5 2hyt 2hzf 2hzk 2hzq 2i02 2i0k 2i0o 2i2q 2i3d 2i3h 2i48 2i53 2i5h 2i5i 2i5r 2i5u 2i6h 2i6v 2i71 2i74 2i7a 2i7g 2i8d 2i8e 2i9w 2i9x 2ia1 2ia4 2iai 2ibd 2ibj 2ibn 2ic7 2icg 2ici 2ict 2icu 2icy 2id3 2id6 2idl 2iel 2ieq 2ig0 2ig6 2ig7 2igi 2ign 2igp 2igx 2iid 2iih 2ija 2ijk 2ik9 2ikb 2ikk 2imd 2imj 2imr 2imz 2in0 2inb 2inu 2ion 2ip1 2ip2 2ipr 2iru 2isb 2isw 2it9 2ite 2iu1 2ium 2iuq 2ivn 2iwa 2iwk 2iwx 2ixd 2iy9 2iyy 2iz6 2j05 2j0a 2j0p 2j13 2j1p 2j21 2j2j 2j31 2j3x 2j5n 2j5s 2j5z 2j6a 2j6g 2j6i 2j6v 2j7j 2j89 2j8g 2j8r 2j91 2j97 2j9a 2j9b 2j9e 2j9o 2ja2 2jb1 2jb7 2jbh 2jbz 2jc5 2jc9 2jcb 2jcn 2nl9 2nlg 2nli 2nm2 2nml 2no7 2noo 2np5 2npn 2npt 2nq3 2nqt 2nrk 2nrt 2ns9 2nsa 2nsf 2nt0 2ntp 2nuo 2nvd 2nve 2nw0 2nw8 2nx4 2nx9 2nxc 2nxf 2nxw 2nyh 2nyi 2nyu 2o02 2o04 2o0a 2o0j 2o0m 2o0q 2o11 2o1a 2o1c 2o1q 2o28 2o2x 2o31 2o3f 2o3s 2o4j 2o4m 2o5v 2o60 2o62 2o6f 2o6l 2o6y 2o70 2o7i 2o8j 2o8q 2o99 2oa9 2oah 2oai 2ob0 2ob5 2obi 2obl 2obp 2oc3 2oc5 2ocg 2od4 2od5 2odh 2oeb 2sli 2src 2ygs 3ezm 3nul 3std 4uag 4ubp 7odc

Resolution 1.8-2.1

2505 files

16vp 1a28 1a44 1a4m 1a64 1a76 1a8r 1aa7 1acc 1agq 1aik 1alu 1amu 1aol 1aoq 1at0 1atz 1avy 1axi 1b12 1b25 1b2r 1b2x 1b4f 1b59 1b5l 1b63 1b6t 1b77 1b9k 1b9n 1be9 1bhd 1bhe 1bqu 1bs1 1bu8 1buo 1bvw 1bwn 1bwp 1byf 1c0i 1c3c 1c3p 1c44 1c7n 1c7s 1c94 1cb8 1ccz 1cgk 1ci4 1ci8 1cjd 1cjw 1cq1 1cq3 1cqn 1cs0 1cs6 1cs8 1ct5 1ct9 1cxl 1cy9 1czs 1d2m 1d2o 1d2t 1d2z 1d3b 1d3h 1d4w 1d6j 1d7k 1d8i 1d9c 1dbx 1dce 1dcq 1dg3 1dg7 1dgm 1dj8 1dkq 1dl5 1dle 1dlj 1dll 1dly 1dmw 1dnl 1dow 1doz 1dp4 1dpj 1dqa 1dqe 1dqs 1dqt 1drt 1dt1 1dtj 1dus 1duw 1dvo 1dvp 1dxe 1dy4 1dy9 1dyk 1dyo 1dzf 1e0b 1e0c 1e2v 1e2x 1e4f 1e5h 1e5q 1e61 1e6i 1e7u 1e7z 1e86 1e99 1eb0 1ebl 1ed1 1edu 1ee8 1eef 1eej 1eem 1ef0 1eg5 1eh6 1ehy 1eib 1ej2 1ejj 1ek9 1ekj 1el5 1el6 1elq 1elr 1em8 1eo6 1eok 1epw 1eq2 1eqf 1es6 1esi 1esj 1eu8 1ex0 1ex2 1ex8 1eyb 1eyq 1ez3 1ezi 1ezj 1f00 1f07 1f0k 1f0n 1f0y 1f1m 1f1s 1f20 1f2p 1f39 1f47 1f4z 1f5m 1f7s 1f7t 1f8a 1f8m 1f9p 1f9r 1fa6 1fao 1fbq 1fc4 1fc6 1fe6 1ff3 1fg4 1fgq 1fhg 1fio 1fkm 1fkn 1fl2 1fmi 1fnl 1fnn 1foa 1fob 1fp1 1fpo 1fpz 1fse 1fso 1fux 1fx4 1fx7 1fyh 1g0r 1g0s 1g15 1g1b 1g1c 1g1j 1g1k 1g1s 1g3k 1g3q 1g4m 1g55 1g5h 1g5t 1g73 1g7s 1g8e 1g8i 1g8l 1g8p 1ga8 1gak 1gcz 1ge7 1gjw 1gl2 1gl4 1gm9 1gn0 1gp3 1gp4 1gpj 1gqe 1gqy 1gr0 1gsj 1gsm 1gsw 1gu1 1gui 1gus 1gux 1gv4 1gvn 1gwo 1gx1 1gxj 1gxo 1gy2 1gyb 1gyg 1gyh 1gyu 1gzl 1h0h 1h1l 1h1m 1h1y 1h2f 1h3f 1h3g 1h3i 1h3n 1h4o 1h4r 1h5w 1h5z 1h64 1h6d 1h6k 1h6u 1h6w 1h70 1h72 1h7c 1h7l 1h7w 1h83 1h8p 1h8u 1h8v 1h9k 1hbk 1hbm 1hdi 1he7 1hg1 1hhs 1hjl 1hn0 1ho1 1hpu 1hq0 1hrk 1hru 1hs6 1huf 1hyu 1hzi 1i1d 1i2a 1i2h 1i36 1i45 1i4y 1i59 1i5q 1i5x 1i6n 1i7b 1i7w 1i81 1i8a 1i9c 1i9g 1i9z 1ia7 1iap 1iaz 1ic0 1ic2 1ie7 1ied 1ig3 1ihg 1ihj 1iiq 1iir 1ijb 1ijt 1ijx 1ik6 1ikg 1iko 1ilw 1imx 1ini 1inn 1io1 1io2 1iq4 1iqv 1itk 1itv 1itw 1iu1 1iul 1iwe 1iwm 1ix4 1ixk 1ixl 1iye 1izm 1izo 1j09 1j18 1j1i 1j1l 1j1t 1j22 1j2t 1j2x 1j3m 1j4r 1j53 1j5p 1j5u 1j5w 1j6p 1j7k 1j7x 1j85 1j9l 1ja1 1jax 1jbk 1jcf 1jd5 1jdp 1je5 1jfl 1jfz 1jg8 1jgt 1jh6 1jhc 1jhf 1jhs 1jju 1jk7 1jl5 1jmv 1jmw 1jp3 1jpe 1jpn 1jpr 1jq3 1jqe 1jr2 1jr7 1js2 1jsd 1jv1 1jvn 1jwq 1jxm 1jyh 1jyj 1jyn 1jzt 1k04 1k0i 1k0w 1k12 1k1b 1k24 1k27 1k2v 1k30 1k32 1k3s 1k3t 1k50 1k51 1k63 1k6d 1k6j 1k6k 1k6z 1k70 1k72 1k97 1k9e 1ka0 1kar 1kbl 1kbq 1kcm 1kcz 1kdo 1ke1 1kep 1kfm 1kfr 1kgn 1khc 1khx 1khy 1kie 1kk1 1kk9 1kl1 1kl9 1kmo 1kna 1knc 1knq 1ko7 1kp8 1kq9 1ksk 1kss 1ktb 1ktg 1ktw 1kux 1kv9 1kw9 1kww 1kzl 1l0h 1l1d 1l2t 1l3i 1l3p 1l4x 1l5b 1l5w 1l7n 1l8b 1la1 1lb6 1lbv 1lc8 1ldf 1lf6 1lfa 1lg2 1lh0 1lhu 1li4 1lj5 1ljo 1lm5 1lm8 1ln0 1lnw 1lr0 1lr2 1lr5 1lr8 1lrz 1lsh 1lsl 1lt1 1lt8 1ltv 1lua 1lur 1lv0 1lvf 1lvm 1lxi 1lxj 1lxy 1ly1 1ly2 1ly8 1lz0 1m03 1m0d 1m0w 1m0z 1m1h 1m1s 1m2w 1m3u 1m48 1m4d 1m4n 1m4r 1m5s 1m6t 1mai 1mb1 1mbm 1mby 1md0 1me8 1mej 1mgw 1mh9 1mhn 1mhw 1mi8 1mij 1miz 1mki 1mky 1ml4 1ml9 1mmi 1moj 1mp9 1mpg 1mpx 1mr7 1mrz 1ms1 1mug 1muu 1mvl 1mw5 1mwm 1mwp 1mww 1mxu 1mz4 1mzb 1mzg 1n06 1n12 1n1b 1n1f 1n2m 1n3b 1n3i 1n4a 1n5i 1n5t 1n5u 1n7h 1n7z 1n81 1n8u 1n9h 1n9l 1n9p 1naa 1nae 1nd4 1nd7 1ne8 1nff 1nfv 1ng5 1ngn 1nh8 1ni9 1nig 1njk 1njr 1nkz 1nlf 1nnw 1np3 1np6 1npn 1npu 1nqn 1nqx 1nrg 1nri 1nrl 1ntf 1nu2 1nu4 1nuu 1nv0 1nvi 1nvk 1nw6 1nxu 1nxz 1ny1 1nze 1nzn 1nzo 1o0w 1o13 1o1x 1o20 1o22 1o2a 1o3x 1o4t 1o4w 1o50 1o58 1o5j 1o5k 1o5u 1o6a 1o6s 1o75 1o7k 1o7z 1o91 1o94 1oa7 1oao 1oap 1ob8 1obb 1obv 1oby 1ocs 1ods 1oej 1oey 1of1 1of3 1ofc 1ofd 1off 1ofm 1ofo 1ofu 1og1 1ogs 1oho 1ohu 1oi4 1oih 1oiz 1oj4 1oj7 1ojh 1ojp 1ok8 1okg 1oks 1ol0 1olt 1olz 1om0 1om6 1omz 1oo0 1ooz 1opk 1oqw 1or7 1ore 1oru 1osd 1otk 1otv 1ov3 1ov8 1ovn 1ow1 1owl 1oxv 1oy3 1oyj 1oyx 1oyz 1oz9 1ozh 1p0k 1p1h 1p2f 1p42 1p4k 1p4m 1p4n 1p52 1p7j 1p7p 1p81 1p8f 1p8x 1pc5 1pcm 1pdv 1pev 1pfz 1pgv 1pie 1pj3 1pj7 1pjh 1pk8 1pl2 1pl3 1pn2 1pno 1pp1 1ppy 1prz 1psw 1pt1 1pt2 1pt7 1pui 1puj 1pvg 1pvl 1pxv 1py0 1py9 1pyu 1pzm 1pzw 1pzx 1q06 1q08 1q0k 1q0p 1q0q 1q0u 1q0z 1q1c 1q2y 1q32 1q3e 1q40 1q52 1q5x 1q63 1q7t 1q8b 1q8c 1q8d 1q8h 1q8i 1q8j 1q8r 1q9u 1qa7 1qak 1qcc 1qcf 1qcn 1qcs 1qdb 1qh3 1qhd 1qj5 1qj8 1qjb 1qkr 1qmh 1qnt 1qnx 1qo7 1qoq 1qoy 1qq6 1qqm 1qr0 1qrl 1qsj 1qsr 1qv6 1qvz 1qw7 1qw8 1qwi 1qwr 1qx4 1qxo 1qy0 1qyr 1qyv 1qz1 1qz9 1qzm 1qzz 1r0v 1r1h 1r1m 1r1u 1r23 1r2d 1r2k 1r31 1r33 1r3d 1r3r 1r4v 1r4x 1r4z 1r54 1r5z 1r6l 1r75 1r7l 1r7o 1r7s 1r86 1r89 1r8h 1r8j 1r8o 1r9c 1r9h 1r9w 1ra4 1rdj 1rew 1rfe 1rg0 1ri5 1ri6 1rid 1rif 1riy 1rjd 1rjq 1rkb 1rkg 1rkv 1rli 1rlj 1ro0 1ro7 1roa 1rp9 1rpj 1rq2 1rqb 1rqp 1rr9 1rsg 1rss 1ruw 1rwc 1rwi 1rwz 1ry9 1ryi 1rym 1rz1 1rz4 1rzy 1s14 1s16 1s21 1s2c 1s2x 1s30 1s4n 1s66 1s68 1s6f 1s7i 1s7m 1s7z 1s8h 1sbz 1sc6 1sc9 1sd4 1sdo 1sdw 1sed 1sef 1sfl 1sgj 1sgm 1sht 1sl5 1smx 1sog 1soi 1sox 1sq9 1sqf 1sqh 1sqw 1sr8 1ss4 1sum 1sur 1sv0 1svd 1svi 1svm 1svv 1sw5 1sw6 1sz0 1sz9 1szo 1szw 1t06 1t07 1t0h 1t0i 1t14 1t15 1t2a 1t2w 1t3i 1t3q 1t5o 1t64 1t6a 1t6g 1t6l 1t6s 1t7q 1t87 1t8h 1t8q 1t8t 1t95 1t9f 1t9m 1t9q 1ta8 1ta9 1tc2 1tc5 1tcz 1td0 1tdg 1tdh 1te5 1te6 1tef 1tff 1tfz 1tg6 1tg7 1thq 1thz 1tiq 1tjb 1tjl 1tjn 1tjv 1tk1 1tk9 1tki 1tkl 1tl9 1tlv 1tm2 1toi 1tq4 1tqn 1tr0 1tr7 1tr9 1tt0 1tt2 1tt6 1tto 1tu1 1tue 1tuh 1tv4 1tvb 1tvf 1tvl 1twi 1twu 1tx2 1txj 1txo 1tz0 1tzj 1tzu 1tzy 1u02 1u09 1u1t 1u20 1u2b 1u2k 1u2w 1u2x 1u2y 1u3y 1u4l 1u56 1u5a 1u5f 1u5g 1u5p 1u5u 1u6d 1u6t 1u6z 1u79 1u7h 1u7k 1u7p 1u94 1u9l 1ua8 1uag 1ual 1ub0 1ub9 1uc4 1uc7 1uc8 1uch 1udv 1udx 1udz 1uec 1uf3 1ufb 1ugn 1uh4 1uhh 1uhv 1uii 1uir 1uj2 1uj4 1ujc 1ujk 1ujn 1uk9 1ukc 1uko 1uld 1umg 1umw 1umz 1uod 1up1 1upg 1upk 1ups 1uq4 1uqt 1urv 1us3 1usi 1usq 1usr 1ut7 1ut9 1utu 1utx 1uw1 1uw4 1uw8 1uwv 1uwz 1ux6 1uxe 1uxo 1uz0 1uz2 1uze 1v0e 1v0z 1v1a 1v1h 1v1t 1v3y 1v4a 1v4v 1v5c 1v5f 1v5x 1v6c 1v6h 1v6z 1v72 1v73 1v74 1v76 1v77 1v7c 1v7l 1v7v 1v84 1v97 1v98 1v9z 1va6 1vaj 1vav 1vax 1vbk 1vc1 1vc4 1vc8 1vch 1vct 1vcv 1vd3 1vdh 1vdk 1ve2 1ve3 1vet 1vf6 1vfq 1vgj 1vj0 1vj7 1vjl 1vjn 1vjy 1vjz 1vk0 1vk2 1vk4 1vk8 1vkb 1vkc 1vkd 1vkh 1vkm 1vku 1vkw 1vky 1vl0 1vl4 1vl5 1vla 1vlg 1vlr 1vm0 1vmd 1vme 1vp4 1vpk 1vpp 1vpr 1vpt 1vpz 1vq3 1vqq 1vqu 1vqz 1vr3 1vr4 1vr6 1vra 1vyb 1vys 1vzw 1vzy 1w03 1w0o 1w1q 1w1y 1w24 1w2f 1w3p 1w42 1w44 1w4r 1w4t 1w4v 1w5f 1w5o 1w6k 1w6l 1w73 1w78 1w79 1w8g 1w8k 1w8s 1w8z 1w94 1w96 1w9a 1w9g 1w9h 1w9y 1wa3 1wa4 1wc3 1wch 1wcx 1wcz 1wd4 1wd5 1wde 1wdg 1wdj 1wdy 1weg 1weh 1wf3 1wg8 1wiw 1wiy 1wj9 1wjg 1wkb 1wko 1wkv 1wlf 1wlj 1wlk 1wlt 1wlv 1wlw 1wm5 1wmg 1wmx 1wn5 1wnh 1wnx 1woc 1wog 1woj 1woq 1wox 1wpb 1wpp 1wq6 1wr2 1wra 1wru 1ws7 1wsb 1wsr 1wtc 1wur 1wv9 1wva 1wve 1wvn 1wvv 1wwb 1wwc 1wwj 1wwr 1wws 1wx1 1wx2 1wxo 1wy1 1wy9 1wyb 1wz3 1wzc 1wzn 1wzo 1wzu 1x0p 1x0r 1x12 1x13 1x1n 1x39 1x3l 1x3x 1x42 1x55 1x6j 1x7n 1x83 1x88 1x89 1x8c 1x8l 1x9z 1xa3 1xb6 1xb9 1xbw 1xbx 1xdf 1xdw 1xe1 1xed 1xeg 1xeq 1xew 1xfk 1xfo 1xg3 1xg7 1xg8 1xge 1xgw 1xhd 1xhk 1xhn 1xio 1xiy 1xiz 1xjc 1xje 1xk4 1xk9 1xki 1xkw 1xly 1xm3 1xmx 1xnf 1xnj 1xo5 1xov 1xq6 1xqk 1xqr 1xre 1xro 1xru 1xs1 1xs5 1xt8 1xti 1xtp 1xtt 1xty 1xu1 1xua 1xud 1xuu 1xuv 1xv2 1xvb 1xvs 1xw4 1xwq 1xwv 1xxq 1xy7 1xzz 1y02 1y08 1y0e 1y12 1y1x 1y28 1y2u 1y3d 1y42 1y44 1y4j 1y4t 1y5e 1y5i 1y6z 1y71 1y79 1y7p 1y88 1y89 1y8t 1y96 1y9a 1y9g 1y9i 1y9q 1y9t 1y9w 1ya9 1yad 1yar 1yav 1ybx 1yby 1ybz 1yc9 1yd1 1ydg 1ye5 1yf9 1yfn 1ygg 1yh3 1yhf 1yhl 1yht 1yi7 1yib 1yir 1yj7 1yjp 1ykd 1yks 1yli 1ylk 1ylq 1ylu 1ym5 1ynf 1yoa 1yod 1yon 1yoy 1ypf 1yqe 1yqg 1yqp 1yqt 1yqw 1yr0 1yr2 1yr7 1yrr 1ys3 1ysd 1yt8 1ytl 1ytv 1yu2 1yun 1yvi 1yw4 1yx1 1yxm 1yxo 1yy7 1yyh 1yyq 1yzf 1yzh 1yzv 1z02 1z05 1z0e 1z0m 1z0u 1z1y 1z3a 1z40 1z45 1z47 1z4e 1z54 1z5b 1z5z 1z6b 1z6u 1z82 1z83 1z84 1z8h 1z94 1z96 1z98 1z9o 1za4 1zah 1zao 1zav 1zb6 1zch 1zcj 1zcz 1zde 1zdr 1zef 1zel 1zgh 1zhh 1zhs 1ziw 1zix 1zjb 1zjc 1zjr 1zkd 1zl2 1zle 1zli 1zlj 1zlq 1zm0 1zm8 1zmq 1zn6 1znw 1zol 1zp4 1zpv 1zsq 1zt3 1zt9 1ztc 1ztd 1zth 1zud 1zuk 1zuo 1zuz 1zv8 1zvz 1zw0 1zwy 1zx2 1zx8 1zx9 1zxx 1zy4 1zze 1zzg 1zzm 1zzs 2a06 2a0k 2a10 2a1f 2a1k 2a21 2a2m 2a33 2a38 2a3y 2a40 2a4a 2a4v 2a56 2a5h 2a5w 2a5z 2a61 2a67 2a6c 2a6r 2a7g 2a7l 2a7n 2a86 2a8j 2a9p 2a9u 2aag 2aan 2aao 2aco 2ae0 2ae8 2aee 2aej 2aev 2af6 2afb 2ahe 2ahu 2aka 2akp 2amh 2amj 2amm 2amu 2amx 2an1 2ane 2ani 2ao9 2ap1 2apl 2apo 2apq 2aq9 2aqj 2aqt 2ar5 2ar6 2arz 2as0 2ate 2atz 2au3 2auw 2avp 2awx 2awy 2axn 2axo 2axr 2azw 2b0c 2b0o 2b18 2b1y 2b2i 2b2n 2b3b 2b3m 2b3s 2b3y 2b4i 2b4j 2b4l 2b4p 2b4v 2b4y 2b67 2b6c 2b6e 2b78 2b7c 2b8i 2b9l 2b9w 2bb6 2bbd 2bbe 2bbh 2be4 2bei 2bek 2ben 2bep 2ber 2bev 2bfw 2bg1 2bgj 2bgr 2bh8 2bhg 2bhp 2bib 2bjb 2bje 2bjr 2bk5 2bl2 2blf 2bm3 2bnl 2bno 2bo4 2boj 2bou 2boy 2bpt 2bqx 2bra 2brq 2bs6 2bs7 2bsf 2bsj 2bti 2btn 2bv5 2bvc 2bvf 2bw1 2bw3 2bwm 2bx6 2bxx 2byh 2byn 2c0n 2c12 2c1d 2c1l 2c1u 2c21 2c2a 2c2i 2c2k 2c2x 2c3m 2c3q 2c41 2c43 2c49 2c4i 2c4x 2c53 2c59 2c5g 2c5k 2c7g 2c7t 2c8m 2c98 2c9e 2c9h 2ca9 2cay 2cb0 2cb5 2cb9 2cbl 2cbm 2ccl 2ccm 2cd9 2cdu 2ce8 2cei 2cfi 2cfk 2cfm 2cft 2cfu 2cgh 2cgl 2cgq 2ch5 2chg 2cho 2ci8 2civ 2cj7 2cjj 2cjp 2ck3 2ckl 2cl0 2cl3 2cl4 2cmj 2cnz 2cu5 2cu6 2cvb 2cvc 2cvj 2cvz 2cw4 2cw6 2cw9 2cwf 2cwn 2cwq 2cwy 2cx1 2cx5 2cx9 2cxc 2cxh 2cxi 2cxo 2cy5 2cyb 2cye 2cyf 2cyy 2cz4 2cz7 2czc 2cze 2d0o 2d0w 2d1c 2d1i 2d1l 2d24 2d28 2d38 2d3n 2d3z 2d42 2d4m 2d4o 2d4x 2d4y 2d58 2d5b 2d5f 2d5l 2d7j 2db7 2dbb 2dbi 2dbs 2dbw 2dch 2dck 2ddb 2ddh 2ddm 2ddt 2ddu 2de6 2dew 2dfa 2dfi 2dga 2dgb 2dh2 2dh4 2di3 2dj6 2djf 2djh 2djz 2dka 2dkh 2dkv 2dm6 2dm9 2dob 2dok 2dp9 2dpm 2dr1 2dr3 2drh 2drw 2ds0 2dsc 2dsj 2dso 2dst 2dsy 2dul 2duo 2dvk 2dvu 2dwk 2dxw 2dy9 2dyo 2dyr 2dzb 2e0a 2e0n 2e1n 2e1z 2e2a 2e3d 2e3i 2e3q 2e3v 2e4m 2e4q 2e56 2e5d 2e5x 2e5y 2e6d 2e6k 2ebo 2eif 2erv 2es3 2es9 2esb 2esn 2ess 2euk 2ev5 2ev9 2eva 2ewb 2ewf 2ews 2ewt 2ext 2exv 2eyn 2f02 2f06 2f20 2f25 2f2h 2f4m 2f4p 2f4w 2f57 2f68 2f6e 2f6m 2f6x 2f7b 2f7f 2f7n 2f7w 2f96 2f99 2f9f 2f9i 2f9r 2f9w 2fa5 2fa8 2fah 2far 2fau 2fav 2fb0 2fb5 2fbh 2fbi 2fbl 2fbo 2fbq 2fbw 2fby 2fcv 2fd4 2fdj 2fdr 2fdu 2fe8 2fea 2fef 2ff4 2ffn 2fge 2fh7 2fhb 2fhq 2fi0 2fi9 2fic 2fim 2fip 2fiu 2fiy 2fje 2fjr 2fk5 2fk8 2fkb 2fl7 2flt 2fmm 2fn0 2fna 2fno 2fo3 2fop 2fp4 2fpd 2fpo 2fpx 2fqe 2fqo 2fqp 2fqy 2fre 2fs2 2fs5 2fsa 2fsh 2fsj 2fsx 2ftq 2ftw 2fu0 2fu4 2fu9 2fuc 2fur 2fuz 2fv7 2fvh 2fw5 2fwt 2fxi 2fxq 2fxv 2fy6 2fya 2fyt 2fyx 2fzp 2fzs 2fzt 2g09 2g0i 2g1a 2g1h 2g28 2g39 2g3a 2g3b 2g3p 2g3w 2g47 2g4j 2g4k 2g4m 2g4p 2g4r 2g5c 2g5d 2g5g 2g5z 2g64 2g66 2g72 2g7c 2g7g 2g7l 2g7z 2g8l 2g9e 2g9f 2g9t 2g9z 2ga1 2ga4 2gaa 2gag 2gaj 2gan 2gau 2gbb 2gce 2gel 2gf4 2gf6 2gfa 2gfh 2gfn 2gfo 2gg8 2ggo 2ggv 2gh0 2gh4 2ght 2gi3 2gia 2gin 2giv 2gjl 2gk4 2gl9 2gm5 2gn4 2gno 2gnq 2go1 2go2 2go7 2gok 2gop 2gpt 2gpy 2gq0 2gqn 2gqr 2gr1 2gr8 2gs3 2gs4 2gsv 2gt1 2gtq 2gu1 2gu2 2guf 2guk 2gum 2guu 2gux 2guz 2gv2 2gv8 2gvi 2gvx 2gvy 2gw8 2gwd 2gwg 2gwh 2gwn 2gz1 2gz7 2gze 2gzm 2h00 2h0b 2h0h 2h0q 2h0u 2h11 2h12 2h1b 2h1c 2h1r 2h1t 2h28 2h2k 2h44 2h4x 2h5f 2h5n 2h6l 2h79 2h7x 2h98 2h9a 2h9f 2h9u 2hbb 2hbj 2hbo 2hbu 2hc9 2hcf 2hci 2hcm 2hcu 2hcv 2hdw 2hdz 2hek 2hey 2hf1 2hf2 2hf9 2hfu 2hh5 2hh6 2hhp 2hhz 2him 2hiq 2hj1 2hjh 2hjn 2hjv 2hka 2hke 2hku 2hl0 2hl8 2hle 2hlh 2hlj 2hls 2hm7 2hmh 2hml 2hnf 2hnu 2ho1 2hp1 2hp4 2hp7 2hpg 2hpl 2hq4 2hq7 2hq8 2hq9 2hqh 2hqq 2hqt 2hqv 2hqy 2hr6 2hra 2hrb 2hrl 2hrv 2hrx 2hrz 2hsb 2hsi 2hsz 2ht9 2hta 2hu7 2huc 2hv6 2hw4 2hwv 2hx1 2hxr 2hxu 2hxv 2hy1 2hy7 2hyu 2hyx 2hz1 2hz5 2hza 2hzf 2hzg 2hzp 2hzq 2hzt 2i02 2i08 2i0z 2i10 2i1q 2i2c 2i2o 2i2w 2i3g 2i3o 2i44 2i47 2i4l 2i52 2i5e 2i5q 2i5t 2i62 2i6d 2i6g 2i79 2i7a 2i7f 2i7n 2i7v 2i87 2i8b 2i9a 2i9c 2i9u 2i9x 2ia2 2iab 2ib0 2ib8 2ich 2ick 2icp 2icx 2id4 2ide 2iej 2iek 2iew 2ifd 2ifr 2ifx 2ig6 2ig7 2ig8 2ig9 2igp 2igt 2ih8 2ihk 2iht 2ihy 2ii0 2ii1 2iia 2iid 2ijh 2ijq 2ik4 2ikk 2iks 2il4 2ilp 2ilr 2im8 2img 2in3 2in6 2in9 2inc 2inu 2iol 2ip1 2ip2 2ipx 2iq6 2iq7 2iqf 2iqj 2irx 2is5 2is9 2ism 2isn 2ist 2isy 2it3 2it9 2itb 2itf 2iu1 2iup 2iuz 2iv1 2iv9 2ivf 2ivx 2ivz 2iw0 2iw2 2iwb 2iwf 2ixd 2ixi 2ixs 2iy9 2iyw 2izz 2j0a 2j0r 2j1n 2j1p 2j1z 2j33 2j3w 2j41 2j4d 2j4t 2j60 2j6y 2j7u 2j7v 2j83 2j8c 2j8f 2j8h 2j91 2j9u 2jay 2jb3 2jbm 2jbv 2jbw 2jc4 2jca 2jcn 2nap 2nlq 2nmu 2nn4 2no0 2no4 2np5 2nq3 2nq5 2nqc 2nql 2nr5 2nrj 2nrv 2ns0 2ns6 2ns9 2nsg 2nsm 2nsq 2nsy 2nt2 2nte 2ntk 2ntq 2nuj 2nv1 2nvw 2nw6 2nw9 2nwh 2nwv 2nx2 2nxo 2nyd 2nyi 2nyk 2nyv 2nz7 2nzc 2nze 2nzh 2nzm 2nzx 2o08 2o09 2o0j 2o0q 2o0y 2o14 2o1b 2o1c 2o1m 2o20 2o28 2o29 2o2a 2o2g 2o2v 2o34 2o38 2o3j 2o3l 2o4d 2o4t 2o4u 2o4v 2o4x 2o57 2o5f 2o5h 2o5u 2o66 2o6a 2o6d 2o6k 2o6l 2o70 2o72 2o78 2o79 2o7h 2o7m 2o7o 2o7t 2o8j 2o8n 2o8p 2o95 2o9a 2oa5 2oaf 2oai 2ob0 2obd 2obe 2obl 2obt 2obv 2och 2ocl 2ocz 2od0 2od6 2oda 2ode 2odf 2odl 2odp 2odt 2odv 2oer 2shp 2sli 2sqc 3kvt 3pro 3thi 5a3h 6std

Resolution 2.1-2.4

1950 files

117e 16vp 1a79 1a8r 1aa6 1ab8 1acc 1ad6 1aj6 1auq 1ax8 1axi 1b27 1b35 1b3u 1b42 1b4b 1b4n 1b5l 1b62 1b74 1b77 1b78 1b8f 1ba3 1bex 1bh2 1bh5 1bi0 1bo4 1bob 1br9 1bwn 1bwp 1c3h 1c4c 1c80 1c9u 1ca4 1cf2 1cjx 1cm0 1cno 1cr5 1cwq 1cwv 1cy2 1cyy 1d0h 1d1j 1d6h 1d6z 1d7k 1dbh 1dbt 1dcq 1dd4 1ddz 1dev 1dhs 1dht 1dkp 1dle 1dli 1dof 1dq8 1drt 1du3 1dvg 1dvk 1dy9 1dyo 1dzr 1e0d 1e1l 1e1o 1e44 1e5e 1e5i 1e5q 1e5r 1e5x 1e7n 1e89 1e8g 1e8z 1e9r 1e9x 1ed1 1edy 1ee6 1ef0 1ega 1ehy 1eje 1ek9 1em2 1em8 1eom 1eou 1ep5 1epu 1eqf 1es8 1ete 1ev7 1evs 1ewh 1ewk 1exs 1exw 1ey2 1eyr 1ezf 1f0m 1f1s 1f1w 1f1z 1f3p 1f4m 1f6y 1f80 1f89 1f8i 1fb8 1fep 1few 1ff2 1fg3 1fio 1fmv 1fno 1fo4 1foh 1fq0 1fs0 1fwk 1fwy 1fx8 1fxv 1fya 1fyz 1fzr 1g0x 1g1c 1g1q 1g2c 1g31 1g41 1g4r 1g5i 1g5r 1g71 1g77 1g7r 1g8p 1g8y 1g9a 1gd8 1ge6 1gg4 1gjr 1gjw 1gk4 1gkz 1gmj 1gml 1gmn 1gp4 1gqh 1gqp 1gt6 1gtg 1gth 1gu3 1gvh 1gw2 1gwc 1gwj 1gxz 1gyw 1h09 1h0k 1h0o 1h0r 1h18 1h19 1h1o 1h2k 1h2t 1h30 1h3g 1h3i 1h3l 1h3o 1h48 1h4m 1h4u 1h4v 1h54 1h5w 1h6c 1h6g 1h6n 1h6p 1h81 1h8g 1h93 1h9g 1h9x 1ha4 1hcu 1he9 1hi9 1hju 1hkf 1hl9 1hm0 1hm3 1hqd 1hqn 1hsk 1htj 1hvv 1hw7 1hxx 1hy5 1i0z 1i1r 1i21 1i4r 1i5n 1i5p 1i5y 1i6x 1i7m 1i8n 1iar 1ib0 1ic0 1icv 1id1 1ifq 1ih7 1ihk 1ihn 1ihu 1ii6 1ii7 1iic 1ik7 1ik9 1im4 1im8 1ion 1ipi 1iq0 1iq8 1iqv 1irj 1is2 1itc 1iu4 1ivv 1ivy 1iwe 1iwp 1ixc 1ixn 1ixz 1iyd 1izo 1j08 1j19 1j1j 1j1l 1j1z 1j2f 1j2g 1j2z 1j32 1j3k 1j3l 1j5y 1j6r 1j6u 1j6v 1j70 1j7n 1j8d 1j8v 1j9g 1j9k 1jad 1jal 1jaz 1jb4 1jcf 1jep 1jfz 1jgs 1jhh 1ji3 1jil 1jl5 1jlh 1jmm 1jmt 1jn9 1joc 1jog 1jph 1jpx 1jqd 1jqp 1js3 1jtd 1jtt 1juq 1jv3 1jvi 1jvn 1jwb 1jx2 1jxh 1jxo 1jyl 1jz6 1jzd 1k0j 1k0w 1k1f 1k1x 1k3b 1k3r 1k4z 1k53 1k5j 1k6m 1k7e 1k9v 1k9x 1kar 1kbi 1kbo 1kbz 1kc7 1kcf 1kdr 1kfi 1kjn 1kk2 1kk9 1kkr 1kmb 1kmk 1kn9 1kna 1ko0 1ko5 1ko9 1kqn 1ksl 1ktc 1ktz 1ku5 1kut 1kuu 1kwc 1kwi 1kwk 1kws 1kya 1kyq 1kyz 1kzl 1kzp 1l0c 1l0s 1l1s 1l1y 1l3a 1l3c 1l5y 1l7o 1l8d 1l8w 1l9k 1l9n 1lbq 1lbx 1lf6 1lg2 1li5 1lnw 1lr8 1lrn 1lrz 1lss 1lt7 1ltq 1lv8 1lva 1lvf 1lvg 1lw3 1lxd 1lxn 1m01 1m13 1m1e 1m1u 1m32 1m4y 1m4z 1m53 1m68 1m72 1m76 1m8f 1m8t 1mau 1mb1 1mb8 1mb9 1mj3 1mjg 1mk7 1mn3 1mn4 1mp0 1mt1 1mu2 1mul 1mvh 1mvn 1mw5 1mwk 1mzr 1mzs 1mzv 1n05 1n0u 1n1a 1n1c 1n1q 1n1v 1n20 1n26 1n2o 1n31 1n4k 1n5v 1n69 1n7g 1n7r 1n7v 1n81 1n8i 1n9k 1n9n 1n9w 1nbp 1nbw 1nd4 1nd7 1ndv 1ne8 1nf2 1nf6 1ng4 1ngk 1ngn 1njf 1nm9 1nml 1nmn 1nmo 1nn4 1nnd 1nnq 1no1 1no4 1now 1npe 1npr 1npt 1nq3 1nqk 1nqw 1nr6 1nt3 1ntg 1nur 1nv2 1nv8 1nvj 1nw7 1nx2 1nx8 1nx9 1ny5 1nye 1nzf 1o0q 1o28 1o3x 1o4z 1o57 1o59 1o5l 1o5o 1o6e 1o73 1o87 1o89 1o9y 1oah 1obc 1obi 1obv 1ocu 1ocx 1odd 1odf 1oee 1of6 1ofu 1ohe 1ohv 1oi8 1oj5 1oji 1ok4 1okc 1oke 1okg 1okr 1omo 1omz 1on0 1onk 1ons 1ooz 1opm 1or4 1or8 1ore 1orj 1os2 1osc 1ot2 1otv 1ouo 1ov9 1ovd 1ow6 1owc 1owo 1ox8 1oxn 1oxu 1oyz 1ozf 1p19 1p1k 1p2x 1p32 1p35 1p3r 1p3w 1p4u 1p4x 1p5s 1p5t 1p72 1p7j 1p8c 1p9e 1p9o 1p9y 1paq 1pdw 1ped 1pfo 1pgu 1phz 1pie 1pix 1pj3 1pj7 1pjh 1pjq 1pju 1pk8 1pkj 1pmo 1pn4 1pno 1po0 1ppw 1ps9 1psu 1pt2 1pt8 1pug 1pv1 1pxu 1pz8 1pzm 1q05 1q0f 1q0h 1q15 1q1s 1q23 1q3k 1q3x 1q4o 1q4q 1q5o 1q65 1q67 1q79 1q7m 1q87 1qam 1qas 1qdn 1qe6 1qf7 1qfh 1qfj 1qg3 1qgr 1qha 1qhl 1qhu 1qiu 1qj9 1qjv 1qk0 1qmd 1qme 1qmh 1qng 1qni 1qo0 1qq6 1qqg 1qs4 1qsm 1qu0 1qvs 1qvv 1qw6 1qyc 1qyn 1qyr 1qzz 1r0w 1r1v 1r22 1r2e 1r2k 1r31 1r53 1r64 1r6b 1r6c 1r6f 1r6t 1r6y 1r8a 1r8g 1r8k 1r94 1r9j 1rb7 1rbz 1rf4 1rhj 1rhy 1ri5 1rid 1rj8 1rjf 1rjr 1rk3 1rl2 1rlt 1rlw 1ro5 1ro9 1rp1 1rpn 1rr7 1rr9 1rsv 1rtw 1rvx 1ry2 1ryd 1rz1 1rz4 1rzq 1s16 1s20 1s2b 1s2m 1s2v 1s2y 1s35 1s3j 1s3l 1s3q 1s3t 1s4c 1s4m 1s4q 1s4u 1s5b 1s5j 1s61 1s6y 1s7h 1s7m 1s7o 1saw 1sb3 1sb7 1sb8 1sbq 1sd0 1sd2 1sdj 1sed 1sfj 1shx 1sij 1siw 1siz 1sji 1sl6 1sl7 1smc 1so2 1sot 1sp3 1sq5 1sqf 1sqi 1sqj 1sql 1stz 1su0 1sv4 1svv 1sw2 1sw6 1syx 1sz0 1sz2 1sz9 1szs 1t0q 1t27 1t29 1t2o 1t2x 1t33 1t36 1t3d 1t3g 1t3l 1t67 1t6d 1t76 1t77 1t8f 1t9b 1td3 1td5 1tdh 1ted 1tf5 1tff 1tfk 1tg2 1tg6 1tgy 1th0 1th8 1tik 1tjc 1tjf 1tju 1tk6 1tk9 1tlb 1tlg 1tll 1tlq 1tmk 1to5 1tq8 1tqd 1tqp 1tr7 1tto 1ttw 1ttz 1tu5 1tue 1tv3 1tvl 1twf 1twl 1tx0 1txu 1ty4 1ty8 1tyo 1tz2 1tz7 1tza 1tzl 1u04 1u0m 1u0t 1u10 1u19 1u20 1u27 1u2e 1u2g 1u2l 1u2m 1u2z 1u41 1u4n 1u5g 1u5w 1u61 1u6i 1u6m 1u7n 1u7z 1u8a 1u9n 1u9t 1ua7 1uam 1uc2 1uc9 1udd 1uds 1ueg 1uf3 1ufa 1ufh 1uhn 1ui5 1uim 1ujz 1ukc 1uko 1ule 1uli 1umi 1ump 1un1 1un2 1uoc 1uoj 1uoq 1uou 1up8 1upr 1usu 1ut0 1ut9 1utc 1uty 1uuh 1uzh 1uzr 1v04 1v1a 1v2a 1v4e 1v67 1v6v 1v8b 1v8d 1v8g 1v9c 1va6 1vay 1vb3 1vb5 1vc9 1vco 1vdx 1ve3 1ve6 1veu 1vf6 1vj1 1vj7 1vjr 1vjx 1vk0 1vk3 1vk6 1vkd 1vkz 1vlh 1vli 1vlm 1vlq 1vlu 1vm6 1vp7 1vpl 1vpq 1vq0 1vq2 1vqr 1vqy 1vrg 1vrn 1vs0 1vym 1vz0 1vzj 1w00 1w03 1w0j 1w1b 1w1p 1w1u 1w2g 1w3s 1w3t 1w48 1w5d 1w5t 1w6f 1w6h 1w6k 1w6t 1w73 1w7k 1w8e 1w8n 1w8w 1w91 1w9c 1w9y 1wa4 1wc5 1wdi 1wdt 1wdu 1wek 1wjg 1wkw 1wl5 1wls 1wm9 1wmg 1wmi 1wmm 1wmr 1wn0 1wn1 1wn3 1wng 1wno 1woo 1wox 1wp6 1wpg 1wqg 1wrb 1wru 1ws9 1wtd 1wud 1wue 1wv8 1wvb 1wvn 1wvt 1wwb 1wwp 1wx0 1wxd 1wxr 1wy0 1wy6 1wy7 1wy9 1wyy 1wz7 1x07 1x0v 1x2g 1x31 1x3e 1x3l 1x3m 1x6m 1x70 1x7f 1x7o 1x7x 1x89 1x8c 1x8l 1x92 1x9z 1xa5 1xah 1xb2 1xbt 1xc6 1xd7 1xdj 1xdy 1xeb 1xeq 1xez 1xg6 1xg8 1xhc 1xho 1xhx 1xja 1xjc 1xjk 1xk5 1xk9 1xks 1xku 1xkv 1xm7 1xma 1xme 1xms 1xmx 1xpj 1xqe 1xqm 1xqr 1xqx 1xr4 1xrx 1xsj 1xtg 1xtn 1xtz 1xu2 1xuv 1xuz 1xvi 1xvt 1xx4 1xx7 1xxl 1xzp 1y0z 1y10 1y13 1y1a 1y1o 1y23 1y25 1y2i 1y2o 1y2x 1y30 1y3t 1y44 1y6h 1y82 1y8j 1y8q 1ya7 1yad 1yav 1yba 1ybm 1ybo 1ybt 1yce 1yco 1yd7 1ydh 1ydl 1yem 1yey 1yf2 1yg2 1yhc 1yhk 1yio 1yiq 1yir 1yis 1yiv 1yjg 1yjm 1yjz 1yk0 1yla 1yln 1yni 1ynu 1yox 1yp2 1yqy 1yra 1yre 1yrq 1yrt 1ys4 1ysj 1yt5 1yta 1yto 1yvn 1ywq 1yy5 1yyq 1yyv 1yz7 1z0x 1z18 1z1n 1z2l 1z4v 1z54 1z59 1z5h 1z6t 1z6u 1z7u 1z7w 1z85 1z94 1z98 1z9m 1z9s 1z9u 1zat 1zax 1zbm 1zbp 1zbq 1zbs 1zbt 1zc6 1zcw 1zd1 1zd3 1zgn 1zhg 1ziw 1zjh 1zjk 1zk8 1zkd 1zkg 1zm0 1zmq 1znh 1zor 1zq7 1zrh 1zro 1zrq 1zso 1ztc 1ztz 1zu0 1zup 1zvp 1zw2 1zww 1zx1 1zx2 1zx5 1zy9 1zyb 1zyo 1zyp 1zz7 2a06 2a0u 2a11 2a18 2a1f 2a1m 2a1u 2a22 2a25 2a2d 2a2o 2a3q 2a3w 2a4n 2a5h 2a5k 2a5v 2a5w 2a6h 2a6m 2a74 2a7h 2a8d 2a9g 2a9u 2a9v 2aam 2ab5 2abq 2ac1 2ac4 2aca 2ae5 2ae6 2aeg 2aej 2af4 2afa 2afh 2ag8 2ahm 2ahs 2ahw 2ahy 2ako 2al6 2alg 2anj 2anq 2anu 2ar3 2ar7 2ark 2aro 2au1 2aua 2aun 2aus 2av9 2avn 2awi 2awy 2ax3 2axn 2aye 2az3 2azo 2azp 2b1r 2b2a 2b2n 2b34 2b3d 2b3r 2b4e 2b4o 2b59 2b5e 2b6c 2b7n 2b7o 2b7s 2b8e 2b8h 2b8w 2b98 2b9c 2b9y 2bb3 2bbc 2bc5 2bcd 2bco 2bcp 2bdd 2bdq 2bdt 2bdv 2bdz 2be3 2be4 2bej 2bg7 2bgc 2bgj 2bh1 2bh7 2bhm 2biw 2bj7 2bjo 2bkp 2bl2 2bll 2bmj 2bmz 2bnx 2bo3 2boa 2bph 2bq8 2bqq 2brq 2bs5 2bs7 2bsd 2bsf 2bu7 2bvc 2bve 2bvl 2bvy 2bvz 2bwc 2bwn 2bwu 2bx6 2bxv 2bxw 2byk 2byo 2bzn 2c0l 2c0t 2c0u 2c0y 2c13 2c1f 2c1q 2c1w 2c1y 2c24 2c26 2c2y 2c36 2c3c 2c3t 2c3u 2c4h 2c56 2c5i 2c5n 2c5u 2c63 2c7i 2c7n 2c7t 2c8d 2c9c 2c9e 2c9o 2c9p 2c9t 2ca4 2ca6 2cb3 2cbl 2ccd 2cda 2ce4 2ce6 2ce9 2ceq 2cex 2cfx 2cg2 2cg4 2cgj 2ch8 2ch9 2chg 2chu 2cj8 2cja 2cje 2cko 2ckw 2clb 2clu 2cmn 2cn4 2cnx 2co2 2co5 2cqt 2csb 2csu 2cu0 2cu2 2cun 2cuy 2cvh 2cvx 2cw6 2cwn 2cwp 2cya 2czg 2czr 2d00 2d0h 2d1p 2d27 2d30 2d3i 2d41 2d44 2d4g 2d4q 2d4r 2d4w 2d4y 2d5l 2d66 2d6y 2d7f 2d8p 2db0 2dbs 2dck 2dcl 2dda 2ddm 2ddz 2dew 2df5 2dfh 2dfi 2dfu 2dg6 2dg7 2dg8 2dgb 2dgj 2dh2 2dj6 2djw 2dkd 2dou 2dps 2dpy 2dqb 2drh 2drj 2drs 2drw 2drz 2dso 2dt5 2dt9 2dti 2du2 2du9 2dup 2dv6 2dvz 2dw4 2dw6 2dx1 2dxb 2dy3 2dys 2dzz 2e0k 2e18 2e1p 2e24 2e2a 2e2g 2e31 2e3j 2e3m 2e3u 2e4u 2e50 2e59 2e5c 2es0 2esh 2esn 2et6 2eta 2etd 2eth 2ets 2eul 2ev4 2evl 2evu 2ewc 2ewe 2exk 2exu 2exx 2ezu 2f06 2f07 2f0r 2f1r 2f20 2f27 2f2f 2f3e 2f3l 2f4e 2f4i 2f4o 2f4z 2f5e 2f5i 2f5j 2f5k 2f5y 2f5z 2f6h 2f6m 2f7c 2f7t 2f7y 2f82 2f84 2f8j 2f8l 2f98 2f9t 2f9z 2fau 2fb0 2fb2 2fbi 2fbk 2fbw 2fbx 2fca 2fdj 2fdo 2fe1 2fel 2ff2 2fff 2ffg 2fg9 2fgc 2fge 2fgg 2fgy 2fhd 2fi0 2fi7 2fiq 2fiw 2fiy 2fji 2fk3 2fk7 2fkg 2flo 2flt 2fm1 2fml 2fmu 2fmy 2fn1 2fn8 2fop 2fp8 2fpk 2fps 2fqg 2fqm 2fsd 2fsi 2fsk 2fsw 2fu2 2fuc 2fuq 2fv5 2fv7 2fvk 2fwy 2fx0 2fxa 2fyw 2fyz 2fz4 2g03 2g09 2g0d 2g1m 2g2p 2g2x 2g39 2g3i 2g3v 2g42 2g47 2g4v 2g6g 2g7l 2g80 2g8e 2g8n 2g8y 2g98 2g9t 2gan 2gbb 2gbo 2gc3 2gcg 2gcl 2gd6 2gd9 2ge3 2ged 2gef 2gek 2gem 2gf2 2gfa 2gfg 2gfi 2gft 2gfv 2ggb 2ghb 2ghr 2ghv 2gjg 2gjn 2gjv 2gk2 2gk3 2gk6 2gks 2gll 2glx 2gm5 2gmm 2gn1 2gn8 2gnn 2gnv 2go1 2go7 2go8 2gpj 2gpw 2gq2 2gr7 2gru 2grv 2gsk 2gtc 2gtg 2gti 2gum 2gv8 2gvm 2gwc 2gwf 2gwr 2gx8 2gyy 2gzx 2h0b 2h0g 2h1a 2h1e 2h1h 2h28 2h2q 2h2u 2h39 2h3n 2h3w 2h4j 2h4l 2h4q 2h4u 2h6b 2h6r 2h7y 2h8h 2h8u 2hay 2hb0 2hbj 2hcn 2hcr 2hcu 2hd3 2he3 2hf0 2hf8 2hgs 2hgv 2hh9 2hhl 2hig 2hj1 2hj9 2hk2 2hkp 2hkz 2hmv 2hnh 2hnk 2ho4 2hoz 2hp4 2hpt 2hqu 2hr3 2hr7 2hs5 2hsn 2htm 2hv2 2hvg 2hvq 2hw1 2hx1 2hxo 2hyj 2hyo 2hyw 2hyz 2hz5 2hza 2hzm 2i0m 2i15 2i1o 2i1s 2i2a 2i2s 2i39 2i3a 2i4o 2i5e 2i5q 2i6q 2i6u 2i6x 2i72 2i79 2i7h 2i7r 2i7v 2i80 2i89 2i9d 2i9v 2i9z 2ia0 2ia2 2iag 2iaz 2ics 2id0 2idh 2idj 2ido 2ied 2iee 2iep 2if8 2ift 2ify 2ig3 2igs 2ihe 2ihv 2ii3 2ii8 2iib 2iii 2iiu 2iiz 2ijg 2ijl 2il5 2ill 2im0 2in4 2in5 2inp 2io6 2ioj 2ipq 2iqc 2iqx 2irp 2isv 2it3 2iu8 2iut 2ivd 2ivq 2iw3 2iw4 2iwc 2iwp 2iwv 2ixb 2iyb 2iyg 2iz1 2iz7 2izy 2j0b 2j1d 2j1k 2j24 2j2c 2j3m 2j3u 2j3v 2j3w 2j40 2j49 2j4g 2j4o 2j4s 2j56 2j58 2j62 2j67 2j6h 2j6p 2j7x 2j85 2j92 2j9l 2ja4 2ja9 2jai 2jaq 2jbj 2jbr 2jbw 2jcd 2jch 2nlk 2nn2 2nn4 2nnc 2nnl 2nnn 2no9 2np3 2npo 2nq2 2nqr 2nr9 2nrw 2ns2 2ns6 2ns7 2nsc 2nsj 2nsm 2nsx 2nt2 2nto 2ntx 2nu8 2nud 2nv2 2nv4 2nvm 2nvp 2nvw 2nwb 2nwu 2nxj 2nxq 2nyk 2nyv 2nyx 2o09 2o14 2o17 2o18 2o1s 2o1u 2o1z 2o27 2o2a 2o35 2o3b 2o3c 2o3i 2o3u 2o4c 2o4g 2o5n 2o5r 2o6k 2o6w 2o7t 2o8s 2o9b 2oa1 2oa5 2oa6 2oaj 2oas 2ob9 2oba 2obb 2obd 2obe 2obg 2obm 2obn 2obr 2ocx 2odm 2odu 2oe1 2oeg 2siv 2std 3amv 5pgm 7mdh 7req 9gaa

Resolution 2.4-2.7

1422 files

1914 1a0p 1a17 1a2v 1aih 1aua 1ax8 1axc 1b09 1b34 1b35 1b3q 1b4a 1b4n 1b6b 1b87 1b89 1bh9 1biu 1bpo 1bvb 1bwr 1byw 1c0m 1c3n 1c3s 1c3x 1c4c 1c5f 1c7o 1c81 1ce0 1cjx 1cjy 1ckm 1cmi 1cp9 1cy0 1d2p 1d6y 1d7a 1d7m 1d9e 1d9u 1dab 1dd4 1dd5 1ddi 1de0 1dej 1dib 1drm 1dwj 1dxf 1dxp 1dxx 1e0d 1e1c 1e1n 1e22 1e2l 1e2z 1e3g 1e3p 1e44 1e4g 1e5l 1e5s 1e6r 1e6v 1e79 1e8z 1e9i 1e9r 1ecx 1ega 1eh8 1ehk 1eia 1eks 1eod 1ep6 1epu 1eqr 1esm 1esq 1evj 1evq 1ewz 1ex9 1exc 1exw 1eyp 1f05 1f12 1f1z 1f33 1f3h 1f3p 1f42 1f4j 1f5a 1f6d 1f89 1f93 1f9c 1fb8 1fc0 1fd9 1fgu 1fhx 1fiq 1fl9 1fno 1foh 1fp4 1fsi 1fst 1fte 1fu1 1fuu 1fv2 1fwr 1fyr 1g1q 1g2v 1g44 1g4p 1g5z 1g6o 1g82 1g96 1g9x 1gcj 1gg2 1gg5 1ggo 1gju 1gjv 1gk1 1gku 1gl3 1gn9 1gp9 1gq7 1gqc 1gr1 1gt7 1gtd 1gu6 1guo 1gws 1gwv 1gxc 1gye 1gyr 1gyt 1gyw 1gz5 1h0n 1h0x 1h21 1h2i 1h2u 1h31 1h3q 1h4v 1h6a 1h8g 1h9y 1ha4 1hc7 1he9 1hi9 1hiz 1hjq 1hk7 1hk8 1hkx 1hl6 1hl8 1hm8 1hno 1hr6 1htz 1hvv 1hxt 1hyq 1i00 1i1r 1i21 1i31 1i4k 1i4o 1i4w 1i5k 1i78 1iay 1ibv 1icv 1id1 1ifq 1ii0 1ije 1ilg 1ipa 1iri 1irx 1is8 1ish 1it8 1iu4 1iuh 1iv5 1iz1 1j0e 1j0i 1j0r 1j0w 1j19 1j2c 1j3u 1j42 1j8v 1j9g 1j9z 1jad 1jal 1jbq 1jd6 1jdi 1jec 1jeq 1jhe 1ji4 1jiu 1jiz 1jjc 1jjh 1jlw 1jmm 1joe 1jof 1jog 1jpl 1jqp 1jqq 1jr1 1js6 1jsh 1jt6 1jvd 1jxi 1jy8 1jyl 1k0b 1k1x 1k2f 1k47 1k6m 1k8q 1k9a 1k9v 1kac 1kbj 1kbw 1kc1 1keu 1key 1kfi 1kht 1khv 1kiy 1kl7 1kn9 1kne 1knx 1kpi 1kql 1kqo 1krm 1ksv 1ktc 1kuy 1kw0 1kxz 1kyw 1kzy 1l1y 1l2f 1l2u 1l5a 1l6m 1l7c 1l8l 1l8q 1l9g 1lb4 1lbq 1lbx 1lcs 1ldi 1li7 1ljm 1lkz 1lnz 1lr9 1lwh 1lxd 1lzw 1m0b 1m0i 1m1j 1m1l 1m4u 1m7e 1m7r 1m8f 1m8n 1m8p 1m9i 1mbz 1mez 1mft 1mg3 1mi7 1mjt 1ml8 1mmf 1ms0 1muk 1mvk 1mwt 1mzo 1n07 1n0e 1n1a 1n1c 1n23 1n26 1n3s 1n4q 1n6e 1n7u 1n80 1n8w 1nay 1nbw 1ncn 1ng3 1nh7 1nhh 1ni0 1ni5 1njj 1njt 1nkn 1nlm 1nlt 1nmt 1no1 1nou 1nqf 1nr9 1nsl 1nt0 1nt3 1nt4 1ntm 1nug 1nvd 1nw3 1nwm 1nws 1nx3 1nx4 1nxk 1ny5 1nye 1o0v 1o12 1o25 1o4u 1o51 1o59 1o5t 1o70 1o7d 1o7f 1o8c 1o8q 1o99 1o9b 1o9j 1odi 1oe0 1ofe 1ofh 1og0 1og2 1og4 1ogl 1ohc 1ojc 1ok6 1okr 1olo 1olp 1om9 1omx 1onl 1ope 1oqd 1ori 1ory 1ot5 1ow7 1ox6 1oy7 1oyv 1oz0 1ozt 1p0e 1p0y 1p16 1p17 1p1i 1p3y 1p4t 1p5j 1p65 1p7y 1p8r 1p9e 1p9r 1p9s 1pc6 1pcx 1pd3 1pd5 1pdk 1peb 1pf5 1pg5 1pi6 1pjs 1pnq 1pnz 1pqw 1ps1 1ps7 1pt9 1pvh 1pvt 1pwh 1pwo 1pyb 1q07 1q09 1q14 1q17 1q19 1q1t 1q1z 1q3h 1q3i 1q5e 1q77 1q8m 1q9s 1qan 1qas 1qbq 1qc6 1qdl 1qfc 1qfg 1qg2 1qgk 1qhx 1qiu 1qlu 1qma 1qme 1qni 1qs2 1qsm 1qu7 1qub 1quq 1qva 1qxa 1qxx 1qyd 1qyi 1qys 1qz8 1r11 1r14 1r1z 1r2g 1r3n 1r4w 1r59 1r61 1r6a 1r6n 1r74 1r76 1r7h 1r95 1r9g 1raj 1rc2 1rcu 1rcw 1rd4 1rd6 1rh1 1ri4 1rjg 1rks 1rlw 1rq5 1rq7 1rqe 1rqi 1rqr 1rv3 1rxx 1rz5 1rzo 1s1t 1s35 1s3m 1s3r 1s4d 1s56 1s5j 1s7w 1s80 1s8o 1s9a 1saz 1sf2 1sf8 1sfj 1sfr 1sj7 1sj8 1sji 1skv 1snk 1so2 1soz 1sq0 1sq3 1sq4 1ss9 1su5 1sui 1suw 1svo 1svu 1syq 1szf 1szq 1t1s 1t3j 1t3u 1t4o 1t5c 1t5j 1t5l 1t5y 1t75 1t77 1t8p 1t8s 1t97 1t9a 1t9k 1tae 1tbx 1td6 1tex 1tf0 1tgo 1th8 1tiy 1tj7 1tjd 1tlb 1tlq 1tmo 1tn0 1to0 1to3 1to6 1tp7 1tq6 1tq8 1tsj 1tuf 1tw2 1tww 1txk 1tza 1u05 1u10 1u12 1u1j 1u2q 1u2r 1u2s 1u3g 1u3t 1u4f 1u5c 1u5e 1u5z 1u6j 1u6m 1u7f 1u7u 1u8s 1u9y 1ua5 1ub2 1ud6 1ue5 1uef 1ueg 1uei 1ufr 1uho 1ui5 1uiz 1ukw 1ulc 1ulj 1ulv 1uly 1um8 1umc 1umi 1umy 1un2 1un8 1uoj 1upc 1upl 1ur0 1url 1urz 1ut4 1uty 1uun 1uur 1ux5 1ux9 1uyj 1uyn 1uyr 1uzd 1v0d 1v0f 1v1b 1v1g 1v26 1v47 1v4k 1v4n 1v7o 1v8b 1v9d 1vbn 1vbv 1vcf 1vde 1vdm 1vdz 1vev 1vgl 1vjr 1vjt 1vk9 1vkj 1vp5 1vp7 1vpa 1vpv 1vpx 1vpy 1vqv 1vqw 1vqy 1vr0 1vrb 1vs0 1vyg 1vyt 1vz4 1w05 1w08 1w0m 1w18 1w22 1w25 1w2a 1w3a 1w3s 1w49 1w51 1w5a 1w5t 1w7m 1w8y 1w90 1w9z 1wa6 1wbn 1wc0 1wd9 1wdf 1wdu 1wdz 1we1 1we5 1wkp 1wmr 1wom 1wpq 1wsc 1wsv 1wtd 1wuu 1wv4 1wvu 1ww1 1ww8 1wwa 1wwh 1wwm 1wxq 1wy5 1wyg 1wyz 1wzw 1x0g 1x2g 1x36 1x77 1x79 1x8m 1x90 1x94 1x9y 1xa8 1xag 1xbn 1xbt 1xdq 1xdt 1xe5 1xea 1xex 1xhb 1xip 1xja 1xjm 1xk5 1xk8 1xko 1xm2 1xm7 1xmm 1xnw 1xp3 1xp8 1xqi 1xrm 1xs4 1xtv 1xu4 1xve 1xvu 1xwr 1xx5 1xzw 1xzx 1y0z 1y19 1y5l 1y62 1y6k 1y7h 1y7o 1y8c 1y97 1y98 1ya0 1ya5 1yau 1ybe 1ybu 1yce 1yco 1yde 1ydw 1yf0 1yf2 1ygz 1yh8 1yis 1yjl 1yk0 1yk9 1ykb 1yla 1ylf 1ynm 1yo6 1yo8 1yp3 1yrg 1yrl 1ysj 1yt0 1yu3 1yud 1yvw 1yw0 1yz3 1yz4 1z0x 1z1e 1z1w 1z26 1z2z 1z4x 1z69 1z6r 1z6z 1z75 1z8f 1z8t 1z8u 1z9m 1z9w 1zat 1zbo 1zbp 1zbx 1zcc 1zdm 1zgs 1zh1 1zh6 1zh8 1zhg 1zi0 1zlp 1zmb 1zmr 1znp 1ztb 1ztp 1ztu 1zu5 1zv4 1zvf 1zwk 1zx3 1zxe 1zxv 1zyo 1zyp 2a0j 2a1s 2a1x 2a5y 2a6a 2a6h 2a6m 2a6t 2a71 2a74 2a85 2a8e 2a8m 2a8s 2a8x 2a91 2a9b 2ab1 2ac2 2acx 2add 2aeh 2af3 2afc 2ag0 2agv 2ahm 2ahy 2ajr 2ajt 2ano 2anu 2aqx 2arh 2ark 2art 2ary 2au1 2auc 2aun 2av8 2av9 2aw5 2awa 2awu 2ax4 2axp 2ayt 2aze 2azm 2b0r 2b25 2b2w 2b3x 2b3z 2b42 2b4a 2b4t 2b5e 2b5o 2b6m 2b71 2b7f 2b81 2b8n 2ba0 2bas 2bbc 2bdt 2be3 2beo 2bfg 2bfr 2bg2 2bgd 2bgh 2bh0 2bh1 2bh9 2bhm 2bho 2bhs 2bhw 2bih 2bix 2bjh 2bkh 2bkn 2bm0 2bm8 2bma 2bmf 2bml 2bn7 2bnf 2bnn 2bnx 2bo6 2bol 2box 2boz 2bpi 2bps 2br8 2brl 2bt1 2bu7 2bvm 2bvu 2bwg 2bwp 2bxj 2bxw 2byk 2byv 2bz0 2c0k 2c10 2c2g 2c2o 2c35 2c3a 2c3o 2c3t 2c4d 2c4v 2c5s 2c5w 2c7f 2c7m 2c8c 2c9a 2ca1 2cb3 2cb4 2cbg 2cbq 2ce6 2ce7 2cfo 2cfx 2cg3 2cg4 2cg5 2cgk 2cgn 2ch1 2chu 2cim 2ckg 2ckq 2clb 2clt 2cmu 2cmv 2cn1 2co1 2coj 2cqz 2csl 2cv9 2cvw 2cw3 2cw8 2cx6 2cx8 2d0v 2d1f 2d1v 2d2a 2d2i 2d30 2d32 2d3a 2d3w 2d40 2d4r 2d5r 2d7e 2d7i 2d7u 2d7z 2db4 2ddo 2de0 2deh 2df7 2dfe 2dg0 2dg2 2dj5 2djw 2dki 2dns 2dpp 2dps 2dpy 2dq0 2dru 2ds7 2dsb 2dsp 2du8 2dub 2dud 2duk 2dv9 2dw7 2dwr 2dx8 2dyc 2dzt 2dzy 2e0w 2e1a 2e20 2e22 2e25 2e2g 2e2x 2e31 2e3c 2e4v 2eua 2eul 2ev3 2evv 2evx 2exx 2f00 2f17 2f1l 2f2f 2f3r 2f41 2f42 2f4l 2f61 2f6k 2f6s 2f8d 2f8j 2f9a 2f9d 2f9z 2fbv 2fdo 2fen 2fep 2fes 2ffi 2ffj 2ffm 2ffs 2fg8 2fh2 2fh5 2fhd 2fhi 2fia 2fji 2fjp 2fk3 2fkd 2fkg 2fkw 2fl1 2fn6 2fp3 2fpn 2fpt 2fqd 2ftp 2fvk 2fw3 2fx0 2fxa 2fxm 2fyw 2fz4 2fzf 2g02 2g04 2g0t 2g18 2g1l 2g2q 2g36 2g3m 2g49 2g4i 2g5h 2g5w 2g6z 2g7r 2g87 2g9i 2ga6 2gdj 2gek 2gez 2gfw 2gh1 2ghp 2ghr 2ghy 2gk6 2gl7 2glp 2gm3 2gm8 2gmh 2gml 2gn2 2gna 2gp4 2gpz 2gqf 2gre 2grj 2grk 2grv 2gs9 2gsc 2gsl 2gtg 2gth 2gv9 2gvh 2gvq 2gys 2gza 2h08 2h0d 2h0i 2h0l 2h0v 2h1a 2h1h 2h1s 2h1y 2h2q 2h2u 2h3r 2h4l 2h56 2h5x 2h63 2h6y 2h89 2h8v 2h9y 2ha9 2hav 2hbm 2hbx 2hcs 2hd3 2hdi 2hev 2hez 2hh7 2hig 2hj0 2hj3 2hjg 2hjw 2hl2 2hma 2hms 2hnv 2ho5 2hoe 2how 2hp2 2hqa 2hqb 2hr2 2hr3 2hr5 2hrh 2hro 2hs0 2ht5 2htb 2hti 2htn 2hu8 2hut 2hv2 2hvb 2hvq 2hwj 2hwk 2hxo 2hyb 2hzm 2i0m 2i15 2i1l 2i1o 2i1v 2i2x 2i3v 2i45 2i46 2i4o 2i5g 2i6e 2i6r 2i6s 2i6x 2i6y 2i75 2i7x 2i8c 2i9p 2iaz 2ic1 2idg 2idx 2if8 2if9 2ifu 2ihc 2iho 2ii4 2iik 2ij4 2ijr 2ikq 2imb 2inn 2io5 2iqc 2iqq 2iqt 2irp 2iua 2ius 2iv0 2ive 2ivp 2iw3 2iw5 2iwl 2ix0 2ixb 2ixj 2ixo 2iy8 2iye 2iz0 2izv 2j08 2j0w 2j3g 2j3s 2j4b 2j5u 2j5v 2j63 2j8s 2j9t 2jad 2jak 2jat 2jby 2jcg 2jch 2nlk 2nly 2nlz 2nmm 2nmp 2nms 2nnn 2no5 2nov 2np0 2nps 2nq2 2nqq 2ns7 2ntd 2ntl 2nu6 2nun 2nuu 2nuy 2nvn 2nwa 2nwb 2nwu 2nxn 2nxq 2nya 2nye 2nyg 2nyq 2nys 2nz2 2nzt 2o03 2o0g 2o0k 2o1e 2o1o 2o1p 2o1s 2o1u 2o1z 2o26 2o2l 2o2t 2o2z 2o3g 2o48 2o4h 2o5a 2o5n 2o67 2o6w 2o7g 2o7l 2o8g 2o8i 2o8r 2o8s 2o98 2o9z 2oaj 2oas 2obk 2obx 2oca 2ocd 2oef 2phm 2shk 3bmp 3caa 3ygs 7mdh

Resolution 2.7-3.0

916 files

1a7a 1ahp 1asx 1av8 1avc 1avo 1b70 1b7b 1b87 1bbu 1bh8 1bnl 1bq3 1brr 1c85 1c8m 1c9l 1cf1 1ci0 1civ 1czz 1d2q 1d6m 1d7m 1dgs 1dib 1diy 1dq9 1dt2 1dtw 1dxq 1e31 1e32 1e51 1e6v 1e9z 1ea6 1ecx 1eia 1eku 1ems 1eqn 1eqr 1evj 1exc 1f02 1f2n 1f45 1f59 1fav 1fg5 1fnh 1fnm 1fo1 1fpy 1fst 1fsz 1ft3 1fu1 1fvh 1fyv 1fyx 1g3l 1g3r 1g8x 1gfw 1gjq 1gjv 1gku 1gl6 1gmo 1gpz 1gt7 1gtl 1gvm 1gxc 1gxk 1gzu 1h0y 1h2i 1h4t 1h7a 1h8k 1h8o 1hcf 1hci 1hes 1hg3 1hkq 1hqf 1hr8 1hxg 1hxu 1hyg 1hzx 1i2d 1i2z 1i49 1i5k 1ias 1iay 1ieq 1ii4 1ijd 1ijg 1ilh 1im2 1iq1 1iqp 1ir6 1is8 1ism 1iun 1iyx 1iyz 1j0c 1j0j 1j5s 1j5t 1j9z 1jbg 1jd6 1jdn 1jee 1jeq 1jfm 1jmu 1jn1 1jn5 1jpy 1jqk 1jro 1ju6 1jwa 1jx0 1jx7 1jyf 1jzr 1k1s 1k1w 1k23 1k3e 1k83 1k88 1k8q 1k90 1ka4 1ka8 1kaw 1kc3 1kca 1kd7 1kez 1kf6 1khw 1ki9 1kkm 1kl2 1kl7 1klf 1kpl 1kq5 1kqg 1kql 1ku9 1kx1 1kx8 1kxj 1kxz 1ky9 1l1o 1l2g 1l8i 1l8o 1l8q 1l8x 1lda 1lkv 1llw 1lm7 1lqd 1lql 1lsx 1ltk 1ltl 1ltx 1lw7 1m1j 1m2v 1m3i 1m3w 1m5y 1m8k 1mc0 1mcz 1me6 1mf1 1mfz 1mi1 1miw 1mkd 1mkj 1mqs 1mr9 1mt5 1mvx 1mz6 1mzo 1n04 1n0e 1n0v 1n25 1n3r 1n4d 1n5o 1n5x 1n6f 1n7j 1n8w 1n9o 1n9r 1naf 1nbi 1ncn 1nh7 1nhy 1ni3 1nju 1nl6 1nlt 1nlz 1no7 1noh 1nqf 1nr9 1nrk 1nsl 1nt0 1nuf 1nvf 1nvw 1nws 1nxk 1o5h 1o7d 1o7l 1o8o 1odu 1of5 1oft 1og0 1og6 1ogk 1ohy 1okq 1on8 1oql 1osg 1ov4 1ovz 1ow8 1ox9 1oxz 1oy0 1oy7 1ozb 1ozy 1p0n 1p16 1p17 1p22 1p50 1p6a 1p73 1p91 1p9w 1peq 1pjl 1pjt 1pn3 1pv2 1pvj 1pwe 1pwu 1q17 1q1q 1q39 1q6w 1q77 1q78 1q9j 1qag 1qao 1qat 1qb3 1qcw 1qe0 1qfc 1qff 1qhm 1qhn 1qj3 1qmf 1qqe 1qs2 1qso 1qub 1qw5 1qx6 1qxs 1qxx 1qym 1qyy 1qz8 1qzf 1r11 1r1l 1r2g 1r3n 1r52 1r5j 1r6z 1r8i 1rfq 1rfu 1rfz 1ri2 1rj2 1rj6 1rqc 1rqd 1rqf 1rqg 1rs4 1rtl 1rwt 1ryy 1s1w 1s28 1s4d 1s4e 1s80 1s9c 1sdd 1sez 1sfr 1shs 1sk3 1sm8 1soj 1sq3 1sq4 1sqv 1srq 1ssd 1sui 1sxg 1szf 1szi 1szq 1t09 1t2l 1t34 1t35 1t5a 1t5e 1t5j 1t5t 1t6i 1t72 1t7s 1t8r 1t97 1t98 1tae 1tbx 1tee 1tf0 1tf2 1til 1tj4 1tko 1tkr 1tlj 1tlt 1tnu 1to3 1tq6 1tt7 1tuy 1tv7 1twz 1tye 1tz9 1u0r 1u12 1u2d 1u5i 1u7v 1u9z 1ud5 1ue8 1ui1 1uiy 1uk0 1ulq 1uqw 1urh 1urj 1urz 1usv 1uu0 1uur 1uvr 1uwy 1v1g 1v1o 1v41 1v53 1v8q 1v9p 1va7 1vbf 1vbn 1vbv 1vci 1ve7 1vea 1vg3 1vk9 1vll 1vq1 1vrs 1vyj 1vyv 1vz6 1w0e 1w1l 1w1w 1w25 1w2e 1w2n 1w2x 1w3b 1w46 1w52 1w7m 1w8q 1wao 1wau 1wcd 1wd6 1wd8 1wfx 1wkk 1wlh 1wnu 1wp0 1wp9 1wqs 1wth 1wuf 1wwh 1wxz 1wye 1x0x 1x2h 1x36 1x3f 1x6u 1x75 1x77 1x8e 1x8k 1x8z 1x9j 1xae 1xal 1xd8 1xdu 1xe8 1xf9 1xhm 1xk8 1xm2 1xog 1xou 1xp4 1xq4 1xqs 1xrq 1xrs 1xsr 1xto 1xtu 1xv8 1xvl 1xwd 1xwi 1xwo 1y01 1y1l 1y47 1y4u 1y56 1y6n 1y7u 1ybf 1yc6 1yci 1ye9 1yf5 1yh8 1yhz 1yj5 1yk9 1ykh 1yl6 1yud 1yv9 1yvl 1yvu 1yw0 1yw1 1ywk 1z0z 1z1f 1z1w 1z25 1z3i 1z4u 1z4w 1z5v 1z6a 1z6r 1z74 1z7e 1z7m 1z81 1z9d 1zd4 1zhi 1zj9 1zlp 1zof 1zp2 1zpu 1zq1 1zr5 1zun 1zvu 1zwl 1zyd 1zyl 1zzh 2a3x 2a4c 2a6e 2a71 2a7r 2a7s 2a7w 2a87 2a8p 2a9q 2aaj 2abr 2ad5 2aet 2af7 2afv 2ahd 2ahz 2aja 2anb 2ao7 2ar0 2avu 2aw2 2aw9 2axt 2axu 2ayu 2b0l 2b0z 2b20 2b2u 2b39 2b3o 2b4m 2b7l 2b9b 2ba0 2bc9 2bct 2bde 2be1 2be7 2beb 2beo 2bga 2bhl 2bhr 2bhv 2bj9 2bma 2bn0 2bnk 2bo8 2bp5 2bps 2br3 2br7 2brw 2bt1 2bt8 2bty 2bvh 2bvt 2bwp 2bwt 2bxr 2bym 2byv 2c08 2c0o 2c11 2c2v 2c35 2c3e 2c3o 2c4c 2c5c 2c8c 2c8n 2c9a 2c9k 2cb6 2cbn 2cdq 2cea 2cfb 2cfq 2cfy 2cg5 2cg8 2cge 2cgz 2ch2 2ch6 2cjb 2ckd 2cl8 2cly 2cnb 2csg 2cv8 2cvu 2cw7 2cwe 2cy9 2cyd 2czh 2d2a 2d2d 2d2q 2d3p 2d3q 2d43 2d74 2d7h 2d7r 2d9q 2dbo 2dcm 2deo 2dff 2dfj 2dft 2dgd 2dh3 2dh5 2di4 2dkf 2dpn 2dpt 2dpw 2dq3 2dud 2dum 2duu 2dvf 2dwp 2dx8 2dxn 2dy5 2e25 2e33 2e48 2e4x 2e67 2erj 2eui 2ewn 2ewo 2ewv 2ex3 2f0z 2f1h 2f1m 2f3m 2f3o 2f66 2f7l 2f83 2f8d 2f8v 2f9j 2fde 2ffv 2fgh 2fgk 2fgs 2fh3 2fie 2fkd 2flz 2fni 2fo4 2fon 2fpb 2fpf 2fpg 2frx 2fu3 2fxm 2fy8 2fyi 2fzf 2fzl 2g04 2g0b 2g0j 2g2z 2g3j 2g6t 2g6z 2g7r 2g96 2g9d 2ga0 2ga6 2gbx 2gdc 2gfy 2ggz 2ghp 2gif 2gk7 2gk9 2glf 2glu 2gm7 2gn6 2gps 2gqf 2gsw 2gta 2gth 2gu0 2guj 2gy5 2gys 2h0a 2h0f 2h0k 2h0r 2h1i 2h1n 2h2y 2h31 2h36 2h3r 2h5e 2h5x 2h63 2h70 2ha9 2haf 2hag 2hbm 2hfz 2hg3 2hg4 2hix 2hj0 2hjm 2hko 2hld 2hms 2hp5 2hpi 2hq5 2hqb 2hr5 2hr8 2hrr 2hs4 2hu2 2hvv 2hwg 2hxg 2hyf 2hzb 2hzs 2i0i 2i14 2i22 2i2l 2i46 2i4m 2i4r 2i4t 2i58 2i5b 2i6s 2i76 2i9b 2ia9 2ibo 2ic1 2idb 2ie3 2if2 2if7 2ihb 2ihw 2ijf 2ijr 2ilu 2inn 2inr 2io5 2ioh 2ip4 2ipc 2irm 2isi 2isq 2iss 2iua 2iub 2ius 2iuu 2iux 2ive 2ivo 2iwe 2iws 2ix3 2ix5 2ixn 2iyi 2iyk 2iyp 2j0j 2j0x 2j25 2j4e 2j4h 2j4r 2j5t 2j69 2j8a 2j9r 2j9t 2jad 2jan 2jas 2jbt 2jbu 2jbx 2nla 2nlz 2nmm 2nmt 2nni 2nvb 2nw4 2nwa 2nys 2nzi 2o03 2o0o 2o1p 2o1x 2o2t 2o3o 2o4h 2o55 2o5a 2o5p 2o6h 2o7g 2o7p 2o8r 2o8x 2o96 2o98 2oap 2obk 2oca 2ocf 2ocp 3bmp 4hb1

Resolution 3.0-3.3

338 files

1ahp 1aip 1aow 1avz 1b44 1bh8 1bl8 1cjv 1ckl 1cx8 1d6m 1d8e 1diy 1e28 1e69 1e9z 1efr 1f3w 1fav 1fl7 1fvf 1fyw 1fzi 1gki 1gmo 1gqq 1gt8 1gxk 1h6v 1hes 1hlg 1hqx 1hr9 1hwy 1hxu 1i3q 1i7x 1i8l 1ihi 1ii8 1ijd 1iqh 1ism 1ixs 1j0k 1j1w 1j5t 1jcg 1jch 1jrp 1jxa 1jyf 1k23 1k39 1ka4 1kiu 1kpl 1kq5 1kyi 1l0v 1l3w 1l7v 1l8i 1llz 1lm7 1ltk 1ltl 1ltr 1lw2 1lx5 1m10 1m5y 1m74 1m8k 1mbv 1miw 1mqn 1mqs 1mr9 1mv5 1mvx 1n21 1n3t 1n4d 1nbi 1nhy 1nj2 1nlz 1nph 1nqh 1ny3 1nyq 1o96 1ogy 1oql 1osg 1ovz 1p5y 1p69 1p75 1p8t 1pci 1peo 1pu4 1pvj 1q7q 1q90 1q9c 1qag 1qat 1qb3 1qi1 1qkc 1qki 1r10 1r8i 1rer 1rfq 1rh5 1rj2 1rlv 1rp5 1rqd 1rts 1s28 1s9c 1sc5 1slh 1sqq 1sxi 1sze 1t5e 1t8b 1t8y 1tly 1tuy 1u2d 1u43 1u9o 1ua2 1ue8 1uk0 1ulq 1urj 1usz 1uwy 1ux4 1uyo 1v1s 1v8j 1vak 1vb4 1vcg 1vg2 1vyv 1w1i 1w5e 1w60 1wa9 1wcd 1wdw 1wup 1x9j 1xa6 1xiq 1xn1 1xp5 1xqg 1xri 1xv8 1y0x 1y11 1y1u 1y5r 1y6e 1y6j 1y7e 1ya8 1ykh 1ypo 1yr3 1yrh 1yt2 1yvk 1yvl 1yw6 1z3h 1z3i 1z6a 1z7e 1z7s 1zd2 1zm2 1zoo 1zp2 1zq1 1ztm 1ztv 1zvu 1zxo 1zy1 1zy2 1zyc 1zye 2a30 2a3x 2a4e 2a7r 2a81 2a87 2a8z 2aez 2afi 2afv 2ahd 2aho 2aj2 2anc 2avu 2aw6 2axt 2ayu 2b12 2b24 2b39 2b4k 2b7l 2b92 2bb5 2bhv 2bj9 2bke 2br7 2bri 2bt8 2bvg 2bxr 2byj 2c2l 2c38 2c57 2c5d 2c6f 2cb6 2cge 2cvt 2cwo 2d7h 2dbt 2ddw 2deo 2df4 2dq3 2dqr 2dwg 2dxl 2dyq 2e49 2erj 2etc 2ewy 2ex3 2f3x 2f9j 2f9q 2f9y 2ffh 2fkj 2fni 2fnq 2fpc 2fpf 2fug 2g0b 2g3k 2g4c 2g6t 2g8g 2ger 2ggz 2gh8 2ghu 2gra 2guj 2gxf 2h1g 2h1n 2h5k 2hae 2het 2hfz 2hj6 2hki 2hpi 2hrt 2hvv 2i5k 2i69 2i76 2ia9 2ice 2id1 2if2 2if7 2ijf 2iou 2irm 2is3 2iu9 2iwh 2j04 2j0k 2j3e 2j5f 2j68 2j87 2j8a 2jbu 2jcl 2ljr 2nn3 2npp 2nrp 2nsi 2nty 2nw4 2nwc 2o0i 2o0o 2o6u 2o7p 2o8x 2o96 2oaq 2oby 2oce 2pda 5r1r

Resolution 3.3-4.0

76 files

1b44 1bsx 1dzl 1ewt 1fb5 1ft2 1fzi 1g3i 1gt8 1gvi 1h1z 1hx9 1js9 1k3v 1l0v 1ll0 1lx5 1m1c 1mbv 1n9s 1na1 1nqg 1nr1 1pv7 1rts 1twh 1u9o 1uaz 1ut2 1ux4 1vb2 1vb4 1vbp 1w39 1wdl 1wl1 1wzx 1xkh 1xri 1y11 1yab 1yz6 1z8l 1zt2 1zye 2a73 2b4k 2b7q 2byq 2c2l 2c39 2c5d 2ch4 2ckj 2cwo 2d3b 2dcu 2e0z 2e32 2e4y 2fcq 2ffl 2fug 2fx3 2g5i 2gd4 2gid 2hqf 2htw 2i2r 2ijd 2ix6 2jbp 2npp 2o9x 3tat
